# Physical Activity, Physical Fitness, and Motor Competence in Children Aged 5–10 Years—A Systematic Review

**DOI:** 10.3390/healthcare14131813

**Published:** 2026-06-23

**Authors:** Paulino Gomes Rosa, Guilherme Eustáquio Furtado, Miguel Jacinto, Sergio José Ibáñez, João Serrano

**Affiliations:** 1 ESECS, Polytechnique University of Leiria, 2411-901 Leiria, Portugal; miguel.s.jacinto@ipleiria.pt; 2 SPRINT—Sport Physical Activity and Health Research & Innovation Center, 6000-084 Castelo Branco, Portugal; j.serrano@ipcb.pt; 3 Portugal Life Quality Research Centre (CIEQV), 2040-413 Rio Maior, Portugal; 4 SPRINT—Sport Physical Activity and Health Research & INnovation Center, Polytechnic University of Coimbra, Rua Dom Joao III—Solum, 3030-329 Coimbra, Portugal; guilherme.furtado@ipc.pt; 5 Applied Research Institute, Polytechnic Institute of Coimbra, Rua da Misericórdia, Lagar dos Cortiços–S. Martinho do Bispo, 3045-093 Coimbra, Portugal; 6 Research Centre in Sports Sciences, Health Sciences and Human Development (CIDESD), 6201-001 Covilhã, Portugal; 7 Facultad de Ciencias del Deporte, Grupo de Optimización del Entrenamiento y Rendimiento Deportivo, Universidad de Extremadura, 10003 Caceres, Spain; sibanez@unex.es; 8 Department of Sports and Well-Being, Politecnic University of Castelo Branco, 6000-266 Castelo Branco, Portugal

**Keywords:** physical literacy, motor skill development, child health promotion, movement behaviours, fundamental movement skills, school-based interventions

## Abstract

Background: Motor competence (MC), physical activity (PA), and physical fitness (PF) are interrelated components of child development, yet evidence on the MC–PF–PA triad in middle childhood remains inconsistent due to methodological heterogeneity. Objective: To synthesise associations between MC, PF, and PA in children aged 5–10 years (2020–2025) and to appraise methodological quality and certainty. Methods: A PRISMA 2020-compliant systematic review (PROSPERO: CRD42024617560) searched six databases (PubMed, Scopus, Web of Science, SportDiscus, SciELO, PsycINFO) from January 2020 to April 2025, updated November 2025. Eligible observational studies in English reported quantitative associations between ≥2 constructs in 5–10-year-olds using validated instruments. Risk of bias was appraised in duplicate with JBI checklists. Quantitative pooling was unfeasible under a pre-specified pooling matrix; associations were synthesised narratively and certainty rated using a GRADE-style framework. Results: Thirteen studies (n = 43–1064; 11 cross-sectional, 2 longitudinal) were included; six were low, five moderate, and two high risk of bias. MC–PA was predominantly positive, especially with objective instruments; MC–PF was consistently positive; PF–PA was less consistent but trended positive longitudinally. Mediation/moderation analyses (k = 3) supported roles for perceived competence, self-efficacy, and PF. Null/negative findings clustered among subjective PA measures and higher risk of bias. GRADE certainty was low-to-moderate. Conclusions: MC, PF, and PA are interconnected in middle childhood, but cross-sectional predominance and instrument heterogeneity preclude causal inference; findings are provisional and should drive longitudinal, methodologically standardised research.

## 1. Introduction

Physical activity (PA), motor competence (MC), and physical fitness (PF) are central components of healthy development in middle childhood [1]. Regular PA in the early years is associated with cardiovascular and skeletal health, cognitive function, self-esteem, and academic performance [2,3], and induces neurobiological adaptations relevant to neuromuscular efficiency [4,5]. MC is defined as the ability to perform a broad range of goal-directed movements with quality, adaptability, and coordination, encompassing locomotor, object control, and stability skills [6]. In the body of evidence relevant to this review, MC is most commonly operationalised through the Test of Gross Motor Development (TGMD-2/3) [7] and the Körperkoordinationstest für Kinder (KTK) [8]. PF refers to health- and skill-related attributes—cardiorespiratory endurance, muscular strength, agility, and flexibility—typically assessed with batteries such as FITNESSGRAM or Eurofit [9]. PA is any bodily movement produced by skeletal muscles that results in energy expenditure, measured either objectively (e.g., accelerometers, heart rate monitors) or subjectively (e.g., PAQ-C, parental questionnaires) [10].

Several frameworks describe the relationships among these constructs. Stodden et al.’s developmental model hypothesises reciprocal interactions between MC and PA, mediated by perceived competence and PF [11]. Complementary perspectives—Harter’s perceived competence model [12], biological maturation models, and bio-banding approaches—emphasise motivational, maturational, and contextual influences. The original developmental model has been critiqued for its limited treatment of contextual variables and for assuming linear causal pathways across developmental stages [13].

Despite this theoretical scaffolding, empirical findings on the MC–PF–PA triad remain inconsistent. Some studies report strong positive relationships [14]; others report weak or even negative associations, particularly in younger children [15]. A primary source of this inconsistency is methodological heterogeneity. Objective and self-reported PA estimates correlate only modestly, and different MVPA cut-points (Evenson, Freedson, Pate) yield different prevalence estimates. MC batteries differ in scoring approach (process- vs. product-oriented) and in the version applied (TGMD-2 vs. TGMD-3, with non-identical skill sets). PF is indexed by health-related and motor-related components that are not interchangeable. These methodological choices materially affect the magnitude and, occasionally, the direction of reported associations, and any synthesis of the literature must distinguish them explicitly.

The 5–10-year age window is a pivotal developmental period. Children consolidate fundamental movement skills, refine coordination, and establish behavioural patterns likely to persist into adolescence and adulthood [16]. Recent research in this window has examined links between body composition, MC, and school performance [17], and has evaluated school-based programmes designed to improve MC in primary school children [18]. Psychological and contextual factors—physical self-efficacy, perceived competence, enjoyment, sex differences, school setting, and family support—emerge as relevant mediators and moderators [19,20,21,22].

Previous systematic reviews have informed the field but each has addressed a distinct question. Burton et al. (2023) [19] synthesised the MC–PA, MC–PF, and MC–psychosocial associations specifically in adolescents (≥11 years). Bao et al. (2024) [23] examined associations between MC and executive functions across childhood and adolescence. Gao et al. (2021) [16] reviewed the MC–PA–health evidence across broad age ranges. None of these reviews focused specifically on the 5–10-year window; none simultaneously examined the three-way MC–PF–PA relationship using both objective and subjective PA measures in this age group; and none mapped formal mediation/moderation pathways within this developmental period. Foundational pre-2020 evidence (Cattuzzo et al. 2016 [13]; Jones et al. 2020 [24]) is used as a benchmark but reflects a measurement and reporting era that predates the routine use of TGMD-3, harmonised MVPA cut-points, and structured mediation analyses. This is the evidence gap the present review addresses.

Accordingly, the aim of this systematic review is to synthesise empirical evidence on the associations between MC, PF, and PA in children aged 5–10 years, using both subjective and objective assessment methods, published in peer-reviewed journals between 1 January 2020 and 28 November 2025. Specifically, the review (i) maps the direction and magnitude of pairwise associations across the four primary outcomes—MC–PA, MC–PF, PF–PA, and mediation/moderation pathways; (ii) appraises the methodological quality of the included evidence using JBI critical appraisal tools; (iii) provides a limited quantitative synthesis where heterogeneity permits, supported by a transparent pooling matrix; and (iv) appraises the certainty of evidence using a structured GRADE-style framework. The review is intended to clarify current knowledge and to generate hypotheses for longitudinal and methodologically standardised research, rather than to support categorical practical recommendations.

## 2. Materials and Methods

### 2.1. Study Design and Reporting Standards

This SR was conducted in accordance with the PRISMA 2020 guidelines [25]. The completed PRISMA 2020 checklist, indicating the section, page, and line numbers of the manuscript in which each item is reported, is provided as Supplementary Materials (Table S1). The protocol was prospectively registered on the International Prospective Register of Systematic Reviews (PROSPERO) under the identifier CRD42024617560. Deviations from the protocol are reported transparently in Supplementary Table S2, with, for each deviation, (i) the conceptual rationale (independent of the review process), (ii) the expected impact on the synthesis, and (iii) the observed impact after the deviation was applied. The previously stated compliance with AMSTAR 2 (A MeaSurement Tool to Assess Systematic Reviews 2) has been withdrawn because the instrument was not formally applied during the conduct of this review.

### 2.2. Eligibility Criteria

Eligibility was defined using the Population, Exposure, and Outcomes (PEO) framework [26], which is appropriate for observational and epidemiological evidence (Table 1). Population: children aged 5–10 years, of both sexes, with no restrictions on socioeconomic status or baseline PA level. Exposure: levels or patterns of PA and, where applicable, MC or PF acting as exposure or intermediate variables. Outcomes: PA (objective or subjective), PF (health- or skill-related components), and MC (locomotor, object control, or stability skills).

Additional inclusion criteria were: (i) Studies assessing the amount or level of physical activity, physical fitness, and motor competence using validated instruments; (ii) studies published in peer-reviewed journals within the last five years; and (iii) studies with full-text availability in English. Exclusion criteria were: (i) Conference abstracts, reviews, dissertations, or theses; (ii) studies without full-text access, even after contacting the authors; (iii) studies involving children with disabilities or chronic illnesses that would prevent participation in physical activity; and (iv) studies not published in English.

The 2020–2025 publication window was chosen to reflect a coherent contemporary measurement and reporting era. Specifically, (i) the publication of the TGMD-3 [7] and its progressive uptake in field studies; (ii) the dissemination of refined MVPA cut-points for accelerometry in childhood (Evenson, Pate, Freedson) and the resulting shift in PA prevalence estimates; (iii) the post-2019 expansion of formal mediation and moderation analyses in this literature; and (iv) the post-2019 reorientation of childhood physical activity research, including COVID-19-related disruptions and recovery. Foundational pre-2020 syntheses (Cattuzzo et al. 2016 [13]; Jones et al. 2020 [24]) are used as benchmarks in the Discussion (Section 4.3) but are not included in the synthesis. The implications of this restriction for the conclusions are addressed in Section 4.5.

Exclusion criteria were: (i) conference abstracts, reviews, editorials, commentaries, theses, and dissertations; (ii) studies without full-text access after contacting authors; (iii) studies involving children with disabilities or chronic conditions that would preclude PA; (iv) studies reporting results only for populations outside the 5–10-year range without age-stratified data; and (v) studies that did not use validated instruments or that did not report a quantitative association (correlation coefficient, regression coefficient, odds ratio, standardised mean difference) between the target constructs.

A per-study eligibility justification, indicating for each of the 13 included studies the construct(s) measured, the specific eligible association extracted, the exact age range, the validated instruments used, the alignment with the review outcome, and the funding source as reported by the primary study, is provided in Supplementary Table S6.

### 2.3. Search Strategy

A systematic search was conducted in six electronic databases: PubMed, Scopus, Web of Science (Core Collection), SportDiscus (EBSCO), SciELO, and PsycINFO (EBSCO). The original search was completed on 11 April 2025, and an updated search using the same strategy—with the date filter extended to the new end date—was performed on 28 November 2025 to capture records published during the review process. The update search yielded 0 additional records. Reference lists of all included studies and of relevant systematic reviews were hand-searched; no grey literature sources were included. No automation tools were used at any stage of study selection.

Search strings combined keywords and controlled vocabulary related to the population (e.g., child, children, schoolchildren, primary school children, elementary school children, preschool children, school-aged, paediatric) and to the target constructs (PA, PF, MC, fundamental movement skills, motor coordination, physical literacy). MeSH ‘Child’ and ‘Child, Preschool’ were used in PubMed; APA Thesaurus terms in PsycINFO; and SportDiscus subject headings where applicable. Search strings were adapted to the syntax of each database. The complete, database-specific search strategies—including verbatim strings, fields searched, Boolean operators, language and date filters, dates of execution, and number of records retrieved per database for both the original and the updated search—are provided in Supplementary Table S3. For illustration, the PubMed strategy is summarised in Table 2. The Boolean search strings used to identify eligible studies are presented in Table 3.

### 2.4. Study Selection Process

**Figure 1:**
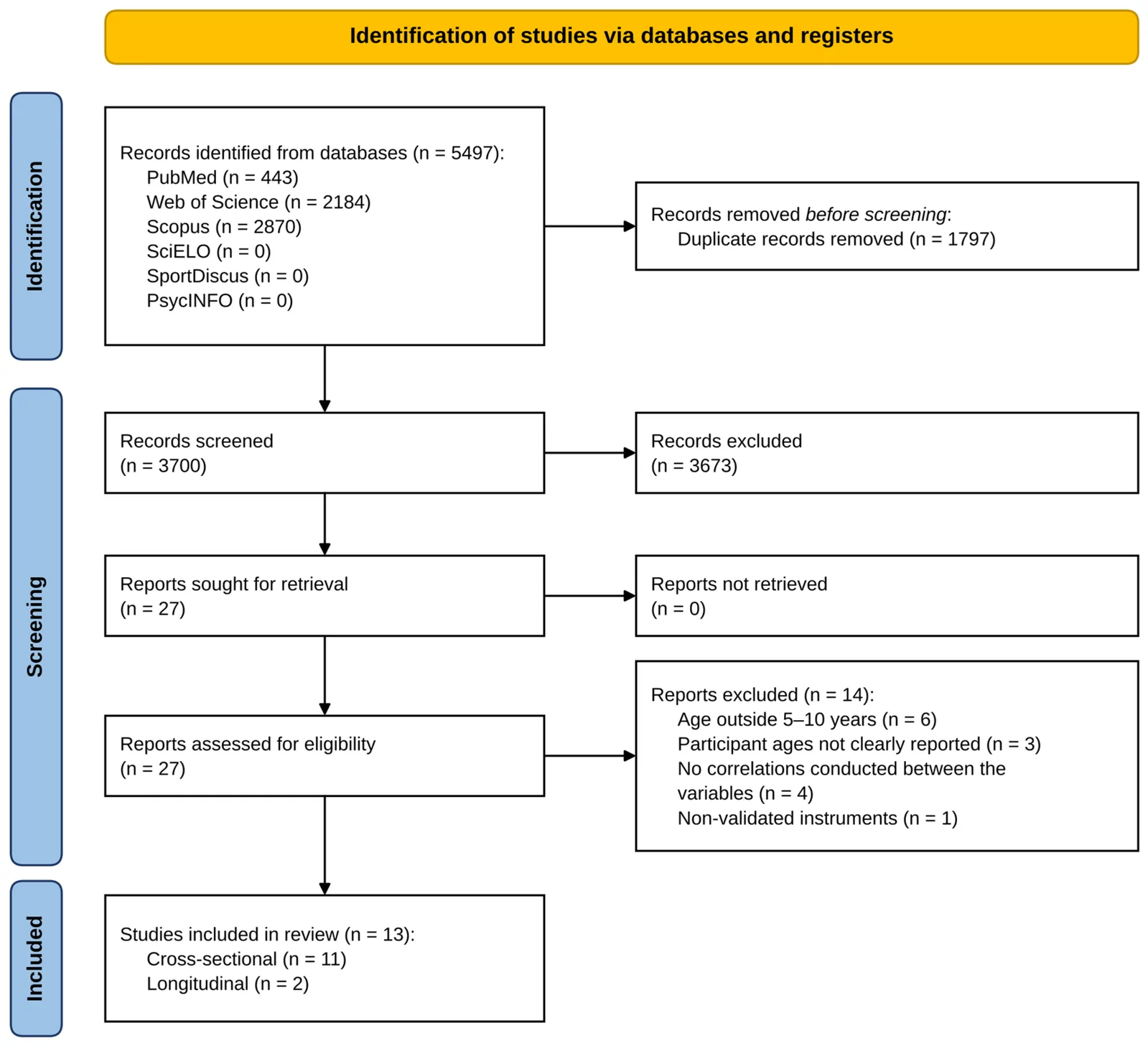
PRISMA 2020 flow diagram illustrating each phase of the search and selection process.

Two independent reviewers (PR and MJ) conducted the search and screening process and verified by a second reviewer (MJ) using a pre-designed Excel extraction sheet. No automation tools were used. Initially, duplicates were removed. Titles and abstracts were then screened based on the predefined eligibility criteria. Full-text articles of potentially relevant studies were assessed for final inclusion. Disagreements were resolved through discussion or by consulting a third reviewer (J.S.) when consensus could not be reached. Inter-rater agreement was calculated at the full-text stage using Cohen’s kappa (κ = 0.83, almost perfect agreement). The selection process is illustrated using a PRISMA flow diagram (Figure 1). The 14 reports excluded at the full-text stage, with study-specific reasons, are listed in Table S4.

### 2.5. Data Extraction

A pre-piloted extraction form was developed in Microsoft Excel. The initial set of fields was piloted on three studies; minor adjustments to definitions of effect metric fields and adjustment set capture were made before extraction proper. Data were independently extracted by one reviewer (P.R.) and cross-checked by a second (M.J.), with a third reviewer (J.S.) arbitrating residual disagreements. Where data of interest were missing or unclear, the corresponding authors of the primary studies were contacted by e-mail; a minimum of two reminders were sent before treating the information as unavailable. The final extraction sheet is provided in Supplementary Table S7.

Variables and rules of extraction (PRISMA 2020 item 10).

Primary variables. The three primary constructs were MC, PA, and PF, each with the operational definition stated in Section 1. Where multiple measures of the same construct were reported, priority was given in the following order: (i) objectively measured outcomes (accelerometer-derived MVPA for PA; validated motor batteries such as TGMD-2/3 or KTK for MC; standardised batteries such as FITNESSGRAM for PF); (ii) the measure most frequently reported in the literature; and (iii) the measure with the most complete reporting (mean, standard deviation, sample size, confidence interval).

Role of variables. For each study, variables were classified a priori as exposure, outcome, mediator, moderator, or covariate, based on the analytical model reported by the primary authors. Claims of mediation or moderation were recorded only where a formal statistical mediation or moderation analysis (Baron–Kenny, bootstrapped indirect effects, structural equation modelling, or interaction terms) had been performed; other indirect pattern descriptions are reported as associational. The three studies with formal mediation/moderation analyses are summarised in the Results.

Effect metrics. The following effect metrics were extracted when available: Pearson or Spearman correlation coefficients (r); unstandardised and standardised regression coefficients (B, β); odds ratios and 95% confidence intervals; and standardised mean differences. Where studies reported several models for the same association, the fully adjusted model was preferred and the adjustment set recorded.

Heterogeneity of instruments. Extracted information explicitly distinguished (a) objective versus subjective PA measurement (e.g., ActiGraph vs. PAQ-C); (b) product- versus process-oriented MC batteries (e.g., TGMD-3 vs. KTK); (c) health-related versus motor-related PF components; and (d) the MVPA cut-points applied (Evenson, Freedson, Pate).

Secondary variables. Covariates and potential confounders considered by the primary studies (age, sex, BMI, maturational status, socioeconomic status, parental PA) were also extracted, together with information on study design, setting, funding source (recorded as ‘not reported’ when ambiguous), and conflicts of interest.

### 2.6. Methodological Quality and Risk of Bias

Risk of bias in the individual studies was assessed independently and in duplicate by two reviewers (P.R. and G.F.) using the Joanna Briggs Institute (JBI) Critical Appraisal Checklist for Analytical Cross-Sectional Studies (8 items) for the 11 cross-sectional studies, and the JBI Critical Appraisal Checklist for Cohort Studies (11 items) for the two longitudinal studies [27]. The JBI tools are validated risk-of-bias instruments, aligned with Cochrane methodological principles and specifically designed for observational designs. The STROBE checklist, referenced in the previous version of this manuscript, is a reporting guideline rather than a validated risk-of-bias instrument and was therefore replaced. The conceptual rationale and observed impact of this change are documented in Supplementary Table S2.

**Figure 2:**
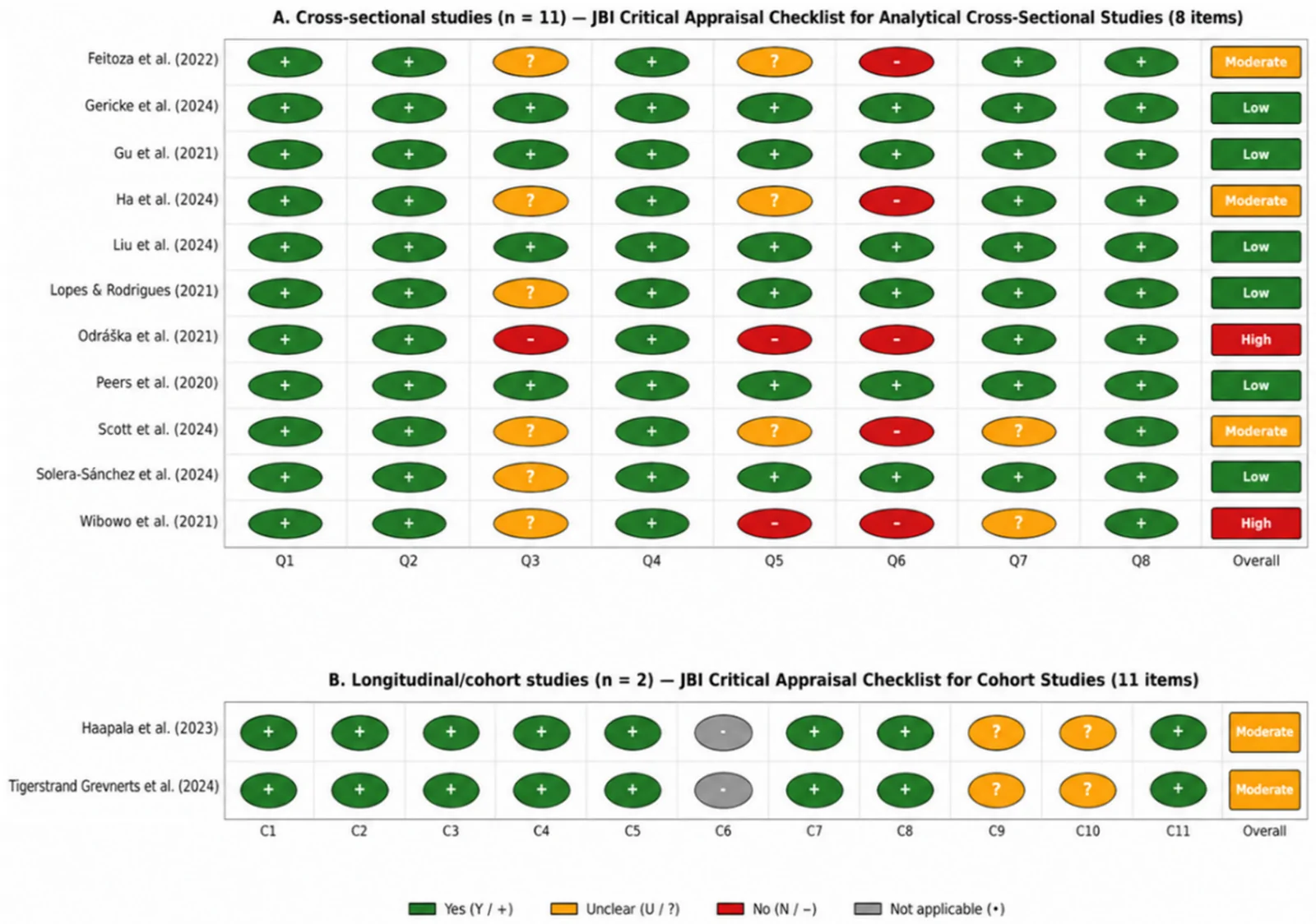
Risk-of-bias assessment across the 13 included studies, based on the JBI Critical Appraisal Checklist for Analytical Cross-Sectional Studies (**A**) and the Cohort Studies checklist (**B**) [28,29,30,31,32,33,34,35,36,37,38,39,40]. Green = yes; yellow = unclear; red = no; grey = not applicable. The right-hand column shows the overall judgement (low, moderate, or high risk of bias).

Each study was rated per item as yes, no, unclear, or not applicable, and an overall judgement of low, moderate, or high risk of bias was reached by consensus between the two reviewers, with arbitration by a third reviewer (J.S.) when needed. Inter-rater agreement at the item level was κ = 0.83 before discussion. No studies were excluded on the basis of risk-of-bias judgement. Item-level ratings, with brief justifications for every ‘no’ or ‘unclear’ judgement, are reported in Supplementary Table S5. The domain-by-domain summary and the traffic light plot is given in Figure 2. The influence of risk of bias on the interpretation of findings is discussed in Section 4.4. The risk-of-bias judgements for each included study are summarised in Figure 2.

To examine whether the distribution of risk of bias varied with the direction of association reported and with the PA measurement modality, we cross-tabulated the JBI overall judgements against these two features (Table 4). The cross-tabulation replaces the post hoc verbal claim made in the previous version of this manuscript and is interpreted descriptively in Section 4.4.

### 2.7. Synthesis Methods

#### 2.7.1. Rationale for Not Performing a Meta-Analysis

We deliberately did not perform a quantitative meta-analysis. To make this decision transparent rather than narrative, we constructed a study-by-study pooling matrix (Supplementary Table S8). For each candidate association—MC–PA, MC–PF, PF–PA, and mediation pathways—the matrix records the number of contributing studies, the construct pairing, the specific instruments used, the reported effect metric, the availability of standard errors or confidence intervals, whether transformation to a common metric (Fisher’s z for correlations) was considered, and the precise reason pooling was rejected for that subset. The minimum threshold for a clinically meaningful pooled estimate was set a priori at three internally homogeneous studies sharing construct, instrument, effect metric, and target population, accompanied by usable variance information.

After applying this pre-specified rule, we identified no subset that met all criteria simultaneously. The four reasons that drove this conclusion, each documented at the per-association level in Supplementary Table S8, are as follows. (i) Clinical heterogeneity. The 13 included studies span recess, physical education, leisure time, and whole-day monitoring across Europe, Asia, North America, and Africa, with sample sizes between 43 and 1064 children and substantial differences in socioeconomic background. (ii) Methodological heterogeneity. MC was assessed with at least four distinct instruments (TGMD-2, TGMD-3, KTK, FITNESSGRAM-aligned batteries); PA combined accelerometry (ActiGraph, Actical, Actiheart) with self- or parent report (PAQ-C, PANIC, PA Enjoyment Scale) and heart rate monitoring; and MVPA cut-points (Evenson, Freedson, Pate) differed across studies, generating non-comparable PA estimates. (iii) Incompatible effect metrics and adjustment sets. Reported estimates included Pearson and Spearman correlations, unstandardised and standardised regression coefficients, odds ratios, and standardised path coefficients from structural equation models, with heterogeneous covariate adjustment. Variance estimates and confidence intervals were not consistently reported, which prevented inverse variance pooling even when effect metric incompatibility could in principle have been handled by transformation. (iv) Insufficient internally homogeneous subsets. For no specific MC–PA, MC–PF, or PF–PA pairing did we identify three or more studies that shared construct, instrument, effect metric, and usable variance information.

We also considered, and rejected, the option of forcing a pooled estimate by transforming reported correlations to Fisher’s z and combining them across instruments (TGMD-2 and TGMD-3 with KTK; objective with subjective PA), as some prior reviews have done. We judged that any such estimate would be uninterpretable: the construct measured by, for example, a TGMD-3 product score plus an Evenson-cut accelerometer is not the same construct measured by a KTK total score plus a self-reported PAQ-C, and a single pooled correlation across both would conceal the methodological signal that the present synthesis is intended to surface. Burton et al. (2023) [19] and Bao et al. (2024) [23] meta-analysed broader heterogeneous evidence in adjacent populations; we agree that this is a defensible choice in some contexts, but in the present 5–10-year evidence base—where instrument choice and PA modality systematically modify the observed direction of association (see Section 3.3 and Section 3.4)—we believe a pooled estimate would obscure rather than clarify.

We acknowledge explicitly that the absence of pooled estimates reduces the methodological robustness of the synthesis relative to a meta-analysis, and we report this in the Limitations (Section 4.5) rather than framing the narrative-only synthesis as a strength. Supplementary Table S8 is provided so that any reader (or future research team) can inspect, association by association, the precise reason pooling was rejected and revisit the decision when additional studies become available.

#### 2.7.2. Narrative Synthesis

All remaining associations were synthesised narratively. Studies were grouped by the four primary outcomes (MC–PA, MC–PF, PF–PA, mediation/moderation) and stratified by (i) objective vs. subjective PA; (ii) product- vs. process-oriented MC batteries; (iii) health-related vs. motor-related PF; (iv) cross-sectional vs. longitudinal design; and (v) low vs. moderate/high JBI risk of bias. For each subgroup, the number of contributing studies and the direction of association (positive, null, negative, mixed) are tallied. Reported statistical estimates were extracted as provided; no imputation of missing summary data was performed.

Pooling was not attempted for the remaining associations because of (a) clinical heterogeneity in setting, socioeconomic background, and geographical region; (b) methodological heterogeneity across instruments and cut-points; (c) incompatible effect metrics with inconsistent variance reporting; and (d) insufficient internally homogeneous subsets. The absence of pooled estimates for these outcomes reduces the methodological robustness of the synthesis and is acknowledged as a limitation in Section 4.5.

#### 2.7.3. Certainty of Evidence

Certainty of evidence was appraised using a structured GRADE-style framework. For each of the four primary outcomes, we rated risk of bias (based on JBI judgements aggregated by outcome), inconsistency (direction and magnitude across studies), indirectness (population, exposure, comparator, outcome), imprecision (sample size and confidence interval considerations), and publication bias (narrative assessment, including evidence of selective reporting, the predominance of significant findings, the absence of unpublished protocols past their planned completion date, and the implications of the English-language restriction). An overall certainty judgement (high/moderate/low/very low) and a brief rationale are provided. The results are summarised in Section 3.

## 3. Results

The study selection process followed the PRISMA 2020 guidelines and is illustrated in the flow diagram (Figure 1). Identification: a total of 5497 records were identified across six databases (PubMed: 443; Web of Science: 2184; Scopus: 2870; SciELO: 0; SportDiscus: 0; PsycINFO: 0). After removing 1797 duplicate entries, 3700 unique records remained for initial screening. Titles and abstracts of the 3700 records were screened based on the eligibility criteria. As a result, 3673 records were excluded. The remaining 27 full-text articles were retrieved and assessed for eligibility. Among them, 14 studies were excluded for the following reasons: (i) participant age was outside the target range (5–10 years); (ii) the age of participants was unclear or not reported; (iii) no correlation analysis was conducted between key variables; and (iv) non-validated instruments were used. After full-text review, 13 studies met all inclusion criteria and were included in the qualitative synthesis. No reports were excluded due to unavailability, and no automation tools were used during the screening and selection process.

### 3.1. General Characteristics of Studies

A total of 13 studies met the eligibility criteria and were included in this SR. All studies investigated children aged 5 to 10 years, with sample sizes ranging from 43 to 1064 participants, and generally included both boys and girls (Table 5). These studies employed primarily cross-sectional designs, with a larger number utilising cross-sectional design [28,29]. A total of two studies used a longitudinal approach [13,24]. The research was geographically diverse, encompassing data from European countries [13,24,28,30,31,32,33], Asia [34,35], North America [36,37], and Africa [38].

The assessment of MC was conducted through validated and standardised instruments in most studies. The Test of Gross Motor Development (TGMD-2 or TGMD-3) was the most frequently used tool, evaluating both locomotor and object control skills [32,34,35,38]. Other studies utilised coordination-based tests such as the KTK—KörperkoordinationTest für Kinder [29], FITNESGRAM [33,36], or movement-based agility, spatial abilities, and jumping tasks [13,30,31].

Levels of PA were measured using both objective and subjective methods. Objective tools included accelerometers [13,24,31,34,36,38]. Subjective assessments were primarily conducted using validated instruments and were employed in the majority of the included studies [24,28,29,30,32,33,37]. One study employed heart rate monitors (e.g., Polar H10) and converted the extracted data to estimate physical activity levels [39].

Despite methodological diversity, most studies reported either direct or mediated associations between MC, PF, and PA levels. However, variations in tools, contexts, and populations contributed to inconsistencies in the strength and direction of these relationships (see Table 5).

### 3.2. Main Results

The 13 included studies explored associations between MC, PA and PF in children aged 5 to 10 years, employing varied methodological approaches and measurement tools (Table 5). Despite heterogeneity in design and outcomes, common patterns and relevant divergences emerged across studies.

Several studies reported positive associations between motor competence and physical activity levels. Positive relationships were found between locomotor and object control skills and MVPA during recess and physical education, as well as between MVPA and motor-related physical fitness in younger children [34,38]. Significant associations were also observed between self-reported physical activity and fundamental movement skills, physical self-efficacy, and perceived motor competence [32]. Additionally, strong correlations were identified between leisure time physical activity and performance in physical fitness tasks, including broad jump and flexibility, suggesting convergence between motor competence and physical fitness domains [33].

Some studies reported weaker or unexpected results. A negative association between MC and PA was identified, diverging from theoretical expectations and potentially explained by the influence of self-perception [30]. A low correlation was observed between motor skills and PA during physical education classes [35]. Additionally, minimal or non-significant associations were found between both subjective and objective measures of MVPA and various components of fundamental motor skills [31].

Regarding physical fitness, several studies supported its role as an independent or mediating variable in the relationship between MC and PA. Health-related physical fitness was shown to exert a stronger direct effect on MC than PA itself [36]. Significant yet negative correlations were also reported between motor performance and both total PA and vigorous PA, suggesting a possible misalignment between motor performance and intensity of activity in some children [24]. Furthermore, consistently meeting PA guidelines at ages 4 and 9 was associated with enhanced motor and muscular fitness performance by age 9 [13].

Psychological and perceptual factors were also identified as significant mediators. Enjoyment and self-efficacy were shown to mediate the relationship between cardiorespiratory fitness and PA [28], while perceived motor competence and physical self-efficacy significantly mediated the association between fundamental movement skills and engagement in PA [32].

Some studies specifically examined sex-related or developmental differences. A perfect mediation effect between MC and PA was found among girls, whereas in boys, the relationship appeared to be influenced by fitness levels [29]. Moreover, locomotor and ball skills were not significant predictors of PA behaviour, indicating potential developmental or contextual limitations in using motor performance as a proxy for activity levels [37].

### 3.3. Pooling Matrix and Rationale for Narrative-Only Synthesis

As specified in Section 2.7.1, we did not perform a quantitative meta-analysis. The decision was supported by a pre-specified study-by-study pooling matrix (Supplementary Table S8) that examined, for each candidate association (MC–PA, MC–PF, PF–PA, mediation pathways), whether at least three studies shared construct, instrument, effect metric, and usable variance information. No subset met all four criteria simultaneously.

The closest candidate subset comprised studies reporting Pearson correlations between objectively measured MVPA (accelerometry) and product-oriented MC batteries (TGMD-2/3) in children aged 5–10 years. Even within this subset, however, accelerometer cut-points (Evenson, Freedson, Pate), epoch lengths, and wear time criteria were not standardised across studies, and several studies did not report the variance estimates required for inverse variance pooling. We considered transforming reported correlations to Fisher’s z and combining them under a random-effects model but judged that the resulting pooled estimate would conflate non-comparable measurement regimes and obscure the very methodological signal—that PA modality and MC instrument modify the observed direction of association—that this review is intended to make visible.

We have therefore retained a structured narrative synthesis, organised by primary relationship and stratified by measurement modality, design, and JBI risk-of-bias category. The per-association feasibility analysis underlying this decision is reported in full in Supplementary Table S8, so that the rationale can be inspected at the level of each pairing rather than accepted as a global narrative claim. The methodological consequence of this choice—namely that the synthesis is less robust than a meta-analysis would have been—is acknowledged in the Limitations (Section 4.5).

### 3.4. Certainty of Evidence (GRADE)

Certainty of evidence was appraised for the four primary outcomes using a structured GRADE-style framework. The results are shown in Table 6.

## 4. Discussion

### 4.1. Summary of Main Findings

Across 13 studies of children aged 5–10 years, the MC–PF–PA triad emerged as an interconnected system of associations. MC and PA were predominantly positively associated, with the strongest and most consistent estimates observed in studies that combined objective accelerometry with product-oriented MC batteries. MC and PF were positively associated across all six studies that examined this pairing; longitudinal evidence from Tigerstrand Grevnerts et al. [29] supported a sustained link between meeting WHO PA guidelines and PF gains. PF–PA associations were positive in cross-sectional and longitudinal studies but showed intensity-dependent variation, with vigorous PA negatively correlated with motor-related PF in one African cohort [34]. Formal mediation/moderation analyses, though restricted to three studies [35,37,40], supported a contributory role for perceived competence, self-efficacy, and PF as psychological and physiological mediators or moderators of the MC–PA pathway.

### 4.2. Synthesis of Evidence

Read in conjunction with the GRADE appraisal (Table 6), the synthesis indicates that the MC–PF association is supported by moderate-certainty evidence—consistent direction and magnitude across studies, low average risk of bias, and reasonable indirectness—albeit limited by small samples and the absence of pooled estimates. The MC–PA and PF–PA associations are supported by low-certainty evidence: positive direction predominates, but inconsistency is meaningful, with the divergence largely attributable to PA measurement modality rather than to substantive disagreement about the relationship. Because no quantitative synthesis was performed, precision is appraised qualitatively from individual-study confidence intervals rather than from pooled estimates. The mediation/moderation outcome is supported by very-low-certainty evidence, given that two of the three formal mediation studies were cross-sectional, that indirectness is high (cross-sectional mediation cannot establish causality), and that imprecision is notable.

An integrative reading of the evidence is consistent with Stodden’s developmental hypothesis [11], in which MC and PA reciprocally develop with PF and perceived competence acting as mediators. However, the exclusively observational evidence base does not support causal claims. Recent challenges to the model’s causal assumptions [13] and complementary frameworks emphasising biological maturation, bio-banding, and dynamic systems perspectives offer additional explanatory levers—particularly for sex- and maturation-related differences such as those reported by Lopes & Rodrigues [37] and Ha et al. [32].

### 4.3. Comparison with the Previous Literature

Previous reviews have addressed adjacent questions but with different age windows or constructs. Burton et al. (2023) [19] meta-analysed MC–PA, MC–PF, and MC–psychosocial associations specifically in adolescents, reporting moderate positive pooled estimates for MC–PA. Bao et al. (2024) [23] focused on MC–executive functions across childhood and adolescence. Gao et al. (2021) [16] reviewed the MC–PA–health evidence across broad age ranges. Compared with these reviews, the present synthesis identifies broadly compatible directions of association in the 5–10-year window, with two specific differences. Firstly, the proportion of studies using objective PA assessment is higher in our 2020–2025 sample than in earlier reviews drawing on pre-2020 evidence, which likely contributes to the more consistent positive direction observed for MC–PA. Secondly, our sample includes two longitudinal studies [29,33] that, while limited in number, point toward a temporally ordered MC–PF link not visible in adolescent-only reviews.

Foundational pre-2020 syntheses such as Cattuzzo et al. (2016) [13] and Jones et al. (2020) [24] reported attenuated or mixed MC–PA associations. The contrast with our findings is most plausibly explained by post-2020 measurement standards: the dissemination of the TGMD-3 and refined MVPA cut-points, and the post-2019 expansion of mediation analyses, have increased the resolution with which these associations can be detected.

### 4.4. Methodological Limitations of the Included Evidence

The synthesis is constrained by methodological features of the primary studies. Subjective PA assessment was associated with attenuated or directionally unstable estimates across the included evidence, as shown by the cross-tabulation in Table 5: the four studies reporting null or negative MC–PA associations [28,31,32,39] all relied on subjective PA, and three of the four were rated moderate or high JBI risk of bias. The pattern is consistent with the well-documented attenuation produced by non-objective PA assessment in this age range—children’s recall and self-reported intensity are noisier than accelerometer outputs, and parental proxy reports introduce further error. We emphasise that this convergence does not establish a causal contribution of risk of bias to the observed direction of association; the analysis is descriptive and the previous, more strongly worded post hoc claim has been removed.

Instrument heterogeneity is a second material limitation. TGMD-2 and TGMD-3 use partially overlapping but non-identical skill sets and scoring; accelerometer MVPA varies with cut-point (Evenson, Freedson, Pate); and PF batteries can index health- or motor-related components that are not interchangeable. Within-construct combinations were therefore not consistently comparable across studies. Confounder adjustment was inconsistently reported (7 of 13 studies omitted explicit confounder sets), which constrains effect size interpretation. Wear time criteria for accelerometry were not always reported transparently. Finally, sex- and maturation-stratified analyses were available in only a subset of studies, leaving a substantive gap in the characterisation of developmentally heterogeneous trajectories.

The cross-sectional predominance (11 of 13 studies) restricts the ability to establish temporal precedence. The positive MC–PA associations reported by Liu et al. [36] and Gericke et al. [34] are equally compatible with an MC → PA pathway, a PA → MC pathway, or co-determination by a third variable such as maturational status. Only the two longitudinal studies [29,33] provide tentative temporal evidence.

### 4.5. Strengths and Limitations of This Review

This review followed PRISMA 2020 with a pre-registered protocol (PROSPERO: CRD42024617560), independent duplicate screening and data extraction, and risk-of-bias appraisal using JBI tools validated for observational designs; we describe these as methodological standards followed rather than as distinctive strengths. The genuine strengths we identify are: (i) the specific focus on a homogeneous and developmentally meaningful 5–10-year window; (ii) the joint examination of the MC–PF–PA triad; (iii) the explicit distinction between objective and subjective PA assessment in the synthesis; (iv) the mapping of formal mediation/moderation pathways and their separation from associational patterns (Table 7); and (v) the transparent, pre-specified justification for narrative-only synthesis (Section 3.4), supported by a study-by-study pooling matrix (Supplementary Table S8) that documents, for each candidate association, the precise reason quantitative pooling was not attempted.

Several limitations should be acknowledged. Firstly, the predominance of cross-sectional designs limits causal inference. Secondly, despite the overall quality, specific JBI domains—confounder identification and handling, and the clarity of subjective exposure measurement—were inconsistently reported. Thirdly, the English-language restriction may have introduced language bias, under-representing evidence from Latin America, francophone Africa, and East Asia; SciELO was included to mitigate this risk, but the restriction remains. Fourthly, instrument heterogeneity prevented quantitative pooling for all four primary outcomes; the absence of pooled estimates reduces the methodological robustness of the synthesis relative to a meta-analysis. Fifthly, the 2020–2025 publication window—although justified by the rapid evolution of measurement tools—excludes pre-2020 foundational studies and may have selectively included evidence post-dating the dissemination of objective measurement standards. Sixthly, publication bias was assessed narratively only, given the absence of a meta-analytical subset; the narrative assessment cannot exclude selective reporting, and the predominance of significant findings in the included evidence is consistent with such selection. Seventhly, the small number of included studies (n = 13) implies that all interpretations should be regarded as provisional. Finally, the geographical representation was skewed toward European and middle- to high-income contexts, limiting the generalisability of the findings.

### 4.6. Implications for Research and Practice

Implications are presented as evidence-graded suggestions rather than categorical recommendations, in keeping with the largely observational and partially cross-sectional evidence base. The synthesis does not support claims that improving one construct will necessarily improve the others; any practical recommendation should be regarded as a hypothesis to be tested by longitudinal and quasi-experimental research.

For research, we identify four concrete priorities. Firstly, longitudinal and quasi-experimental designs beginning in early childhood and extending through adolescence should be prioritised, with at least three measurement occasions to support trajectory modelling and cross-lagged effects. Secondly, measurement standardisation is essential: report the TGMD version used and the scoring approach; report accelerometer brand, epoch length, wear time criteria, and MVPA cut-points (preferably Evenson in this age range); and co-report objective and subjective PA measures with reliability statistics. Thirdly, integration of psychological and contextual factors—perceived competence, enjoyment, self-efficacy, school climate, family support—in multivariable models, ideally with directed acyclic graphs specifying the assumed causal structure, will help move the field beyond bivariate associations toward theoretically informed models. Fourthly, evaluations of integrated programmes that target both motor and psychological development, using pre-registered protocols and blinded outcome assessors, would address the gap in causal evidence; sharing anonymised participant data and analytical code would further facilitate subsequent individual-participant-data meta-analyses.

For practice, the available evidence is consistent with—but does not establish—the usefulness of school-based programmes that combine structured motor skill instruction with developmentally appropriate and enjoyable PA opportunities. Where feasible, objective PA monitoring during PE and recess can support intervention tailoring and standardised outcome evaluation. Policymakers should note that attenuated associations in studies with weaker measurement have implications for investment in rigorous programme evaluation.

## 5. Future Research Directions

To advance understanding of the motor competence–physical fitness–physical activity “triangle,” future research should prioritise longitudinal and quasi-experimental designs beginning in early childhood and extending into adolescence. The use of standardised and objective tools—for example, accelerometer combined with wearable heart rate sensors and validated motor test batteries—will enhance the comparability of results across populations. Integrating psychological variables (such as perceived competence, enjoyment, and self-efficacy) and contextual factors (including school infrastructure, play environments, and cultural climate) within multivariable models will help identify causal mechanisms.

In parallel, greater standardisation of outcome measures and more consistent reporting of key methodological elements—such as bias control, sample size estimation, and data transparency—will improve study quality. Finally, evaluations of integrated programmes that target both motor and psychological development, especially through robust, pre-registered protocols, should be considered a priority.

## 6. Conclusions

This systematic review synthesised the available empirical evidence on the associations between motor competence (MC), physical fitness (PF), and physical activity (PA) in children aged 5 to 10 years, thereby directly addressing the research question. The findings demonstrate that these three constructs form an interdependent system, in which higher motor competence is generally associated with greater engagement in physical activity and improved fitness. In turn, higher fitness levels appear to reinforce the acquisition of motor skills and sustained participation in PA. This reciprocal relationship is amplified by psychological factors, such as perceived competence and self-efficacy, but may weaken in contexts—school, family, or community—where opportunities for active play are limited.

While the high methodological quality of most included studies strengthens the reliability of the evidence, the predominance of cross-sectional designs, heterogeneity of instruments, and limited representation of diverse populations restrict causal inference and comparability. These gaps highlight the need for longitudinal, standardised, and culturally inclusive research to inform early interventions and policies that promote equitable and sustainable physical literacy from childhood.

## Figures and Tables

**Table 1:** Construction strategy for review preparation based on PEO statement.

Acronym	Information	Concept Adapted to Current Study
P	Children aged 5–10 years	Includes both boys and girls from diverse racial and ethnic backgrounds, with no restrictions based on socioeconomic status or prior physical activity (PA) level [27]
E	Physical Activity and Motor Competence Development	Encompasses structured and unstructured physical activities aimed at improving motor competence, fundamental movement skills, and physical fitness [22]
O	PA, Physical Fitness, and Motor Competence	Examines indicators such as PA levels (measured objectively or subjectively), components of physical fitness (e.g., endurance, strength), and motor competence (e.g., coordination, movement skills) [12]

Notes: The PEO framework is particularly suitable for observational, epidemiological, or cohort studies that analyse relationships between exposure and outcomes in the specific population.

**Table 2:** Worked example of the database-specific search strategy (PubMed).

Search Component	PubMed Syntax (Executed 11 April 2025; Re-Run 28 November 2025)
Population	(child[Title/Abstract] OR children[Title/Abstract] OR schoolchildren[Title/Abstract] OR “primary school”[Title/Abstract] OR “elementary school”[Title/Abstract] OR preschool[Title/Abstract] OR “school-aged”[Title/Abstract] OR pediatric[Title/Abstract] OR youth[Title/Abstract] OR kid[Title/Abstract]) OR (“Child”[MeSH] OR “Child, Preschool”[MeSH])
Exposure	AND (“Physical Activity” [Title/Abstract] OR “Motor Activity”[MeSH])
Outcomes	AND (“Physical fitness” OR “Physical capacity” OR “Motor competence” OR “Movement competence” OR “Physical competence” OR “Motor development” OR “Motor skill” OR “Motor ability” OR “Movement skill” OR “Motor coordination” OR “Object control” OR “Manipulative skill” OR “Locomotor skill” OR “Stability skill” OR “Athletic competence” OR “Athletic skill” OR “Motor proficiency” OR “Fundamental movement skill” OR “Movement ability” OR “Motor performance” OR “Movement performance” OR “Movement proficiency”)
Filters	English; 2020/01/01:2025/11/28

Note: full database-specific strategies (Scopus, Web of Science Core Collection, SportDiscus, SciELO, PsycINFO), with verbatim strings, field tags, filters, dates of execution, and per-database record counts, are provided in Supplementary Table S3.

**Table 3:** Boolean search strings for identifying studies on physical activity, physical fitness and motor competence in children.

Search Number	Search String
5	(child OR youth OR kid) AND (“Physical Activity”) AND (“Physical fitness” OR “Physical capacity” OR “Motor competence” OR “Movement competence” OR “Physical competence” OR “Motor development” OR “Motor skill” OR “Motor ability” OR “Movement skill” OR “Motor coordination” OR “Actual competence” OR “Object control” OR “Manipulative skill” OR “Locomotor skill” OR “Stability skill” OR “Athletic competence” OR “Athletic skill” OR “Motor proficiency” OR “Fundamental movement skill” OR “Movement ability” OR “Motor performance” OR “Movement performance” OR “Movement proficiency”)

**Table 4:** Distribution of JBI risk-of-bias categories by direction of association and PA measurement modality.

PA Measurement Modality	Direction of Association	Low RoB (n)	Moderate RoB (n)	High RoB (n)
Objective (accelerometer/HR)	Positive	4	1	0
Objective	Mixed/partial	0	2	1
Objective	Null/negative	0	0	0
Subjective (self/parent report)	Positive	2	0	0
Subjective	Mixed/partial	0	1	0
Subjective	Null/negative	0	1	1

Counts represent unique study × outcome contributions; some studies contribute to more than one outcome and may appear more than once. RoB = JBI risk-of-bias overall judgement. PA = physical activity. HR = heart rate monitoring.

**Table 5:** Summary of study characteristics, methodological approaches, and key findings.

Author	Design	Country	Participants	Measuring Instruments	Results
Haapala, E. A. et al. (2023) [33]	Longitudinal analysis	Finland	N = 189 (81 girls [43%], 108 boys [57%])	Total PA assessed using the PANIC questionnaire filled by parents; MVPA assessed with a combined heart rate and movement sensor (Actiheart^®^); motor performance via 10 × 5 m shuttle run	Significant correlation between motor performance and total PA: r = −0.17 *. Correlation between motor performance and vigorous PA: r = −0.27 *
Ha, T., Fan, X., & Dauenhauer, B. (2024) [32]	Cross-sectional	USA	N = 82, 51.2% male, mean age 10 ± 0.861 years	PAQ-C; TGMD-3	Locomotor and ball skills were not significant predictors of PA behaviour (F(2, 79) = 2.028, *p* = 0.138)
Odráška, L. et al. (2021) [38]	Cross-sectional	Slovakia	N = 91 (45 boys, 46 girls, age 8.37 ± 1.63)	Leisure PA time (questionnaire); HRF via FITNESSGRAM; standing broad jump, 10 m shuttle run, sit and reach, sit-ups	Significant correlation between leisure PA and motor skill performance: broad jump r = 0.664 **, agility run r = −0.695 **, flexibility r = 0.737 **, sit-ups r = 0.636 **
Wibowo, R. et al. (2021) [39]	Cross-sectional	Indonesia	N = 43 (23 boys, 20 girls)	TGMD-2; PA measured with Polar H10 heart rate monitor	Low correlation between fundamental skills and PA level during PE classes (r = 0.203)
Solera-Sanchez, A. et al. (2024) [40]	Cross-sectional and longitudinal	UK	N = 383 children, mean age 10.0 ± 0.5 years	CVRS via KIDSCREEN-10; PA via Physical Activity Enjoyment Scale	Self-efficacy and enjoyment of PA acted individually as mediators in the relationship with CRF (*p* = 0.105)
Gu, X. et al. (2021) [30]	Cross-sectional	USA	N = 342 (156 girls), M = 8.40 years	FITNESSGRAM, PE metrics, PACER, Actical accelerometers	Direct effect of HRF on FMS significant: βlocomotor = 0.33, βball = 0.35, stronger than PA to FMS (β = 0.21, *p* < 0.001)
Feitoza, A. H. P. et al. (2022) [28]	Cross-sectional	UK	N = 379, 54.9% boys, mean age 8.2 ± 1.7	PA (questionnaire), MC (locomotor/object skills), perceived MC (pictorial scale)	Unexpected negative relationship between MC and PA (authors justify with *p* and r values)
Peers, C. et al. (2020) [35]	Mediation analysis	Ireland	N = 860, 47.7% female, mean age 10.9 ± 1.16	PA Self-Efficacy Scale, TGMD-3, Pictorial Scale of Perceived Movement Competence, PA (PACE+)	Self-reported PA positively associated with PSE, FMS, PMSC. FMS and PSE had significant positive relationships with PA (R^2^ = 0.03 to 0.09, *p* < 0.001)
Gericke, C. et al. (2024) [34]	Cross-sectional	South Africa	N = 299 (150 boys, 149 girls), mean age 6.83 ± 0.96	BIA, HRPF test battery, TGMD-2, ActiGraph GT3X	Moderate-to-vigorous PA positively associated with HRPF, MRPF and some motor skills. Significant associations: vigorous PA and MRPF (r = −0.36, *p* < 0.001), MVPA and motor skills (r = 0.13, *p* < 0.001)
Liu, D. et al. (2024) [36]	Cross-sectional	China	N = 322 (163 boys, 159 girls), M = 8.12, SD = 1.22	TGMD-2, ActiGraph GT3X-BT accelerometers	Locomotor skills positively related to MVPA during long (B = 1.063) and short recess (B = 1.502); object control skills positively related to MVPA during long recess (B = 1.244) and PE lessons (B = 1.171)
Lopes, V. P. & Rodrigues, L. P. (2021) [37]	Cross-sectional	Portugal	N = 1064 (530 girls), mean age 7.87 ± 1.17	KörperkoordinationTest für Kinder, 50-yard dash, 1-mile run/walk, standing long jump, PA questionnaire	Perfect mediation seen in girls; in boys, MC to PA relation conditioned by PF levels
Scott, J. et al. (2024) [31]	Cross-sectional	UK	N = 182 (85 boys), ages 7–8	MVPA levels (objective and subjective), FMS, spatial ability	Subjective PA correlated with total locomotion (0.213 *), object manipulation (−0.129), stability (−0.002), FMS score (0.029); objective MVPA correlated with locomotion (0.122), object manipulation (−0.043), stability (0.100), FMS score (0.073)
Tigerstrand Grevnerts, H. et al. (2024) [29]	Longitudinal	Sweden	N = 217 (114 boys, 103 girls)	PA via ActiGraph; fitness via shuttle runs, grip strength, long jump	Meeting PA guidelines (at ages 4 and 9) significantly associated with better motor fitness (−0.76 s, *p* < 0.001) and muscle fitness (+4.6 cm, *p* < 0.001) at age 9

Notes: PA = physical activity; MVPA = moderate to vigorous physical activity; MC = motor competence; PF = physical fitness; TGMD-2/3 = Test of Gross Motor Development, 2nd/3rd Edition; assesses locomotor and object control skills; FITNESSGRAM = a battery of physical fitness tests evaluating cardiovascular endurance, muscular strength/endurance, flexibility, and body composition; PACER = Progressive Aerobic Cardiovascular Endurance Run; a shuttle run test measuring aerobic capacity; ActiGraph, Actical, Actiheart = types of accelerometers used to objectively measure physical activity intensity and duration; Polar H10 = chest strap heart rate monitor connected to mobile apps for real-time monitoring of heart rate during physical activity; KIDSCREEN-10 = questionnaire assessing health-related quality of life in children; PAQ-C = Physical Activity Questionnaire for Children; a self-reported measure of general physical activity levels; PSE = physical self-efficacy; the belief in one’s ability to perform physical tasks; PMSC = perceived movement skill competence; self-perception of motor skill ability; HRPF = health-related physical fitness; encompasses cardiovascular, muscular, and flexibility components; MRPF = motor-related physical fitness; fitness components related to motor skills such as agility and coordination. Correlations are presented as r values; *p* values indicate statistical significance. * very week, negative statistically significant correlation; ** moderate to strong statistically significant correlation.

**Table 6:** GRADE-style certainty of evidence for the four primary outcomes.

Outcome	k (n Participants)	Risk of Bias	Inconsistency	Indirectness	Imprecision	Publication Bias	Certainty
MC–PA	11 (≈3800)	Serious	Serious (direction varies with measurement)	Not serious	Not serious	Suspected (English-only; positive findings predominate)	Low
MC–PF	6 (≈2000)	Not serious	Not serious	Not serious	Serious (small samples)	Suspected (narrative)	Moderate
PF–PA	5 (≈2300)	Not serious	Serious (intensity-dependent)	Not serious	Serious	Suspected (narrative)	Low
Mediation/moderation	3 (≈2300)	Serious (2/3 cross-sectional)	Not serious	Serious (cross-sectional mediation)	Serious	Suspected (narrative)	Very low

Notes: k = number of contributing studies. Certainty levels: high/moderate/low/very low. The publication bias column reflects a narrative assessment, including evidence of selective reporting, the predominance of significant findings, and the implications of the English-language restriction (Section 4.4).

**Table 7:** Studies with formal mediation or moderation analyses (k = 3).

Study	Proposed Mediator/Moderator	Statistical Method	Indirect Effect Formally Tested?	CI for Indirect Effect Reported?	Design	Authors’ Interpretation
Solera-Sánchez (2024) [40]	Self-efficacy and PA enjoyment as mediators of CRF–HRQoL	Mediation regression (single-mediator models)	Yes	Yes (study reports *p* = 0.105 for individual mediation effect)	Cross-sectional + longitudinal	Self-efficacy and enjoyment partially mediate CRF–HRQoL
Peers (2020) [35]	Physical self-efficacy (PSE) and perceived movement skill competence (PMSC) as mediators of FMS–PA	Path analysis/mediation models with bootstrapped indirect effects	Yes	Yes	Cross-sectional	Partial mediation of FMS–PA by PSE and PMSC
Lopes & Rodrigues (2021) [37]	PF as mediator (girls)/moderator (boys) of MC–PA	Mediation/moderation regression with sex-stratified models	Yes	Partially (study reports significance, CI not always given)	Cross-sectional	Perfect mediation by PF in girls; moderation in boys

Notes: Cross-sectional mediation analyses (applicable to Solera-Sánchez and to the cross-sectional component of Peers and Lopes & Rodrigues) are compatible with multiple generative models and do not establish causal relationships. PSE = physical self-efficacy; PMSC = perceived movement skill competence; FMS = fundamental movement skills; CRF = cardiorespiratory fitness; HRQoL = health-related quality of life.

## Data Availability

No new data were created or analyzed in this study.

## Supplementary Materials

The following supporting information can be downloaded at: https://www.mdpi.com/article/10.3390/healthcare14131813/s1, Table S1: PRISMA 2020 Checklist; Table S2: Deviations from protocol; Table S3: Database-specific search strategies; Table S4: Studies excluded at full-text screening (n = 14). Not possible; Table S5: JBI item-level risk-of-bias ratings; Table S6: Per-study eligibility justification; Table S7: Final extraction sheet (13 studies, 32 outcome rows); Table S8: Pooling matrix and per-association rationale.

## Abbreviations

Abbreviation	Definition
AMSTAR 2	A MeaSurement Tool to Assess systematic Reviews, version 2
BIA	Bioelectrical Impedance Analysis
BMI	Body Mass Index
CI	Confidence Interval
CRF	Cardiorespiratory Fitness
FMS	Fundamental Movement Skills
GRADE	Grading of Recommendations, Assessment, Development and Evaluations
HRF	Health-Related Fitness
HRPF	Health-Related Physical Fitness
JBI	Joanna Briggs Institute
KTK	Körperkoordinationstest für Kinder
MC	Motor Competence
MRPF	Motor-Related Physical Fitness
MVPA	Moderate-to-Vigorous Physical Activity
OR	Odds Ratio
PA	Physical Activity
PACER	Progressive Aerobic Cardiovascular Endurance Run
PANIC	Physical Activity and Nutrition in Children (questionnaire)
PAQ-C	Physical Activity Questionnaire for Older Children
PEO	Population, Exposure, Outcomes (framework)
PF	Physical Fitness
PMSC	Perceived Movement-Skill Competence
PRISMA	Preferred Reporting Items for Systematic Reviews and Meta-Analyses
PROSPERO	International Prospective Register of Systematic Reviews
PSE	Physical Self-Efficacy
RoB	Risk of Bias
TGMD-2/3	Test of Gross Motor Development, 2nd/3rd edition
WHO	World Health Organization
