# Correction: Rosa et al. Physical Activity, Physical Fitness, and Motor Competence in Children Aged 5–10 Years—A Systematic Review. *Healthcare* 2026, *14*, 1813

**DOI:** 10.3390/healthcare14213756

**Published:** 2026-11-04

**Authors:** Paulino Gomes Rosa, Guilherme Eustáquio Furtado, Miguel Jacinto, Sergio José Ibáñez, João Serrano

**Affiliations:** 1 ESECS, Polytechnique University of Leiria, 2411-901 Leiria, Portugal; miguel.s.jacinto@ipleiria.pt; 2 SPRINT—Sport Physical Activity and Health Research & Innovation Center, 6000-084 Castelo Branco, Portugal; j.serrano@ipcb.pt; 3 Portugal Life Quality Research Centre (CIEQV), 2040-413 Rio Maior, Portugal; 4 SPRINT—Sport Physical Activity and Health Research & INnovation Center, Polytechnic University of Coimbra, Rua Dom Joao III—Solum, 3030-329 Coimbra, Portugal; guilherme.furtado@ipc.pt; 5 Applied Research Institute, Polytechnic Institute of Coimbra, Rua da Misericórdia, Lagar dos Cortiços–S. Martinho do Bispo, 3045-093 Coimbra, Portugal; 6 Research Centre in Sports Sciences, Health Sciences and Human Development (CIDESD), 6201-001 Covilhã, Portugal; 7 Facultad de Ciencias del Deporte, Grupo de Optimización del Entrenamiento y Rendimiento Deportivo, Universidad de Extremadura, 10003 Caceres, Spain; sibanez@unex.es; 8 Department of Sports and Well-Being, Politecnic University of Castelo Branco, 6000-266 Castelo Branco, Portugal

## Text Correction

In the original publication [1], there was an error in the Results section, paragraph 1. The number of databases and two counts of the study selection narrative were inconsistent with the Abstract, with Section 2.3 Search Strategy and with the PRISMA flow diagram in Figure 1, whose values are the arithmetically consistent ones; in addition, one of the four reasons for exclusion shown in Figure 1 was missing from the text.

A correction has been made to the Results section, paragraph 1:

The study selection process followed the PRISMA 2020 guidelines and is illustrated in the flow diagram (Figure 1). Identification: a total of 5497 records were identified across six databases (PubMed: 443; Web of Science: 2184; Scopus: 2870; SciELO: 0; SportDiscus: 0; PsycINFO: 0). After removing 1797 duplicate entries, 3700 unique records remained for initial screening. Titles and abstracts of the 3700 records were screened based on the eligibility criteria. As a result, 3673 records were excluded. The remaining 27 full-text articles were retrieved and assessed for eligibility. Among them, 14 studies were excluded for the following reasons: (i) participant age was outside the target range (5–10 years); (ii) the age of participants was unclear or not reported; (iii) no correlation analysis was conducted between key variables; and (iv) non-validated instruments were used. After full-text review, 13 studies met all inclusion criteria and were included in the qualitative synthesis. No reports were excluded due to unavailability, and no automation tools were used during the screening and selection process.

## Error in Figure

The “Records excluded (n = 3673)” box of Figure 1 contained a residual editing sentence that should not appear. The corrected Figure 1, in which this sentence has been removed and the labels have been harmonised with the text, appears below; all numerical values are unchanged.

The authors state that the scientific conclusions are unaffected. This correction was approved by the Academic Editor. The original publication has also been updated.

## Figures and Tables

**Figure 1:**
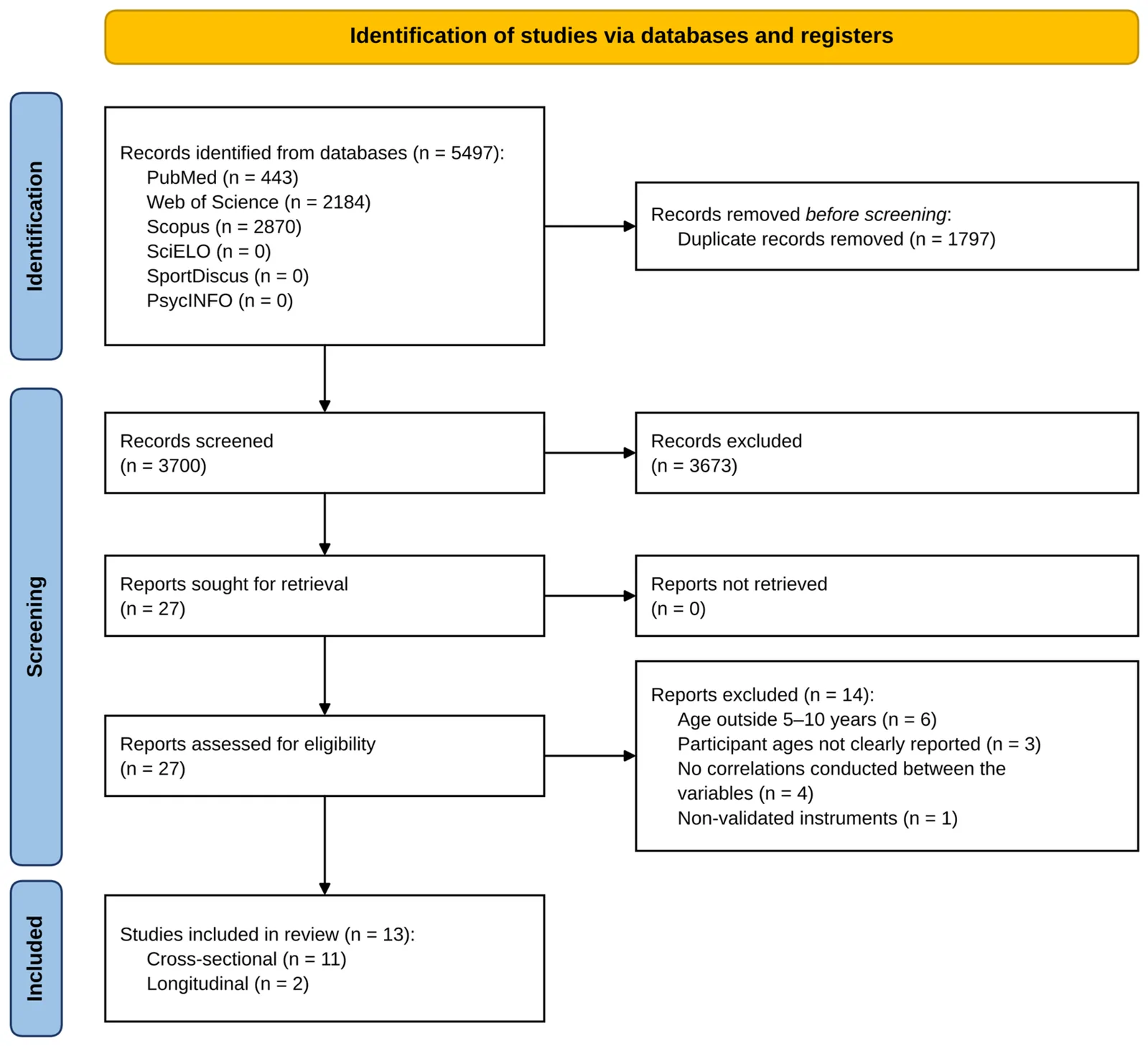
PRISMA 2020 flow diagram illustrating each phase of the search and selection process.
