# Supplementary material for: Physical Activity, Physical Fitness, and Motor Competence in Children Aged 5–10 Years—A Systematic Review
Source: Healthcare (Basel). 2026 Jun 23;14(13):1813. doi: 10.3390/healthcare14131813 (PMC13361801; doi:10.3390/healthcare14131813)
Supplement: Supplementary file 1 [file healthcare-14-01813-s001.zip › healthcare-4198458-supplementary.pdf]

Supplementary Materials

Table S1. PRISMA 2020 Checklist.

PRISMA 2020 checklist mapped to the revised manuscript. Item descriptors quoted from Page MJ et al., *BMJ* 2021;372:n71 (CC BY 4.0).

Item	PRISMA 2020 descriptor	Section, page, line range in manuscript
**TITLE**		
1	Title — Identify the report as a systematic review.	Title page; p. 1
**ABSTRACT**		
2	Abstract — See PRISMA 2020 for Abstracts checklist.	Abstract, p. 1, ll. 5–29
**INTRODUCTION**		
3	Rationale — Describe rationale for the review.	Section 1, p. 2, ll. 33–83
4	Objectives — Provide explicit statement of objective(s) or question(s).	Section 1, p. 2, ll. 84–99
**METHODS**		
5	Eligibility criteria — Specify inclusion and exclusion criteria.	Section 2.2, p. 3–4, ll. 113–149
6	Information sources — Databases and date last searched.	Section 2.3, p. 4–5, ll. 150–173; Table S3
7	Search strategy — Full search strategies.	Section 2.3, p. 4–5; Table 2; Table S3
8	Selection process — Methods used to decide eligibility.	Section 2.4, p. 5–7, ll. 174–188
9	Data collection process — Methods used to collect data.	Section 2.5, p. 7–9, ll. 189–227
10a	Data items: Outcomes — List and define all outcomes.	Section 2.5 (Primary variables), p. 7–8, ll. 189–215
10b	Data items: Other variables — Other variables for which data were sought.	Section 2.5 (Secondary variables), p. 8–9, ll. 215–227
11	Study risk of bias assessment — Methods used.	Section 2.6, p. 9, ll. 228–246
12	Effect measures — Specify effect measure(s).	Section 2.5 (Effect metrics), p. 8, ll. 200–214
13a	Synthesis methods: Eligible studies — Processes to decide eligibility.	Section 2.7, p. 9–12, ll. 247–321
13b	Synthesis methods: Prepare data — Methods to prepare data.	Section 2.5, p. 7–9; Section 2.7.2, p. 11
13c	Synthesis methods: Tabulation/visualisation.	Section 2.7.2, p. 11, ll. 299–314
13d	Synthesis methods: Synthesis methods.	Section 2.7.1, p. 9–11, ll. 248–298
13e	Synthesis methods: Heterogeneity.	Section 2.7, p. 11; Section 3.4, p. 18–19
13f	Synthesis methods: Sensitivity.	Section 2.7.3, p. 11, ll. 315–321; Section 3.5
14	Reporting bias assessment.	Section 2.7.4, p. 12, ll. 322–332; Section 4.4
15	Certainty assessment.	Section 2.7.4, p. 12, ll. 322–332; Table 6
**RESULTS**		
16a	Study selection — Results of search and selection.	Section 3.1, p. 12, ll. 346–370; Figure 1, p. 7
16b	Study selection — Cite excluded studies, explain why.	Section 3.1, p. 12; Table S4
17	Study characteristics — Cite included studies and characteristics.	Section 3.2, p. 13–16; Table 3, p. 15

Item	PRISMA 2020 descriptor	Section, page, line range in manuscript
18	Risk of bias in studies.	Section 3.3, p. 16–18, ll. 428–459; Figure 2, p. 16; Table S5
19	Results of individual studies.	Section 3.2, p. 13–16; Table 3; Table S7
20a	Results of synthesis: Number of studies.	Section 3.2, p. 13–16; Section 3.4, p. 18–19
20b	Results of synthesis: Statistical synthesis.	Section 3.2; Section 3.4 (narrative-only with rationale)
20c	Results of synthesis: Heterogeneity.	Section 3.4; Table 5; Table S8
20d	Results of synthesis: Sensitivity.	Section 3.5, p. 19–21, ll. 482–508
21	Reporting biases.	Section 4.4, p. 23–24; Section 4.5 (limitation vi)
22	Certainty of evidence.	Table 6, p. 20
**DISCUSSION**		
23a	Discussion: Interpretation.	Section 4.1, p. 21; Section 4.2, p. 22; Section 4.3, p. 22–23
23b	Discussion: Limitations of evidence.	Section 4.4, p. 23–24, ll. 564–590
23c	Discussion: Limitations of review processes.	Section 4.5, p. 24–25, ll. 591–621
23d	Discussion: Implications.	Section 4.6, p. 25–26, ll. 622–649
**OTHER INFORMATION**		
24a	Registration and protocol — Registration information.	Section 2.1, p. 3 (PROSPERO: CRD42024617560)
24b	Registration and protocol — Where the protocol can be accessed.	Section 2.1, p. 3
24c	Registration and protocol — Amendments.	Section 2.1, p. 3; Table S2
25	Support.	Funding, p. 28, l. 739
26	Competing interests.	Conflicts of Interest, p. 29
27	Availability of data, code and other materials.	Data Availability Statement, p. 28; Tables S1–S8

Table S2. Deviations from Protocol.

For each deviation from the PROSPERO-registered protocol (CRD42024617560), the table reports the conceptual rationale, the expected impact on the synthesis, and the observed impact.

Deviation	Conceptual rationale	Expected impact on synthesis	Observed impact after deviation
Replacement of STROBE by JBI for risk-of-bias appraisal	STROBE is a reporting guideline and does not provide validated criteria for risk-of-bias appraisal of observational studies. The JBI Critical Appraisal Checklists for Analytical Cross-Sectional Studies (8 items) and Cohort Studies (11 items) are validated risk-of-bias instruments aligned with Cochrane methodological principles and specifically designed for observational designs.	Transition from a single "compliance percentage" to a transparent low/moderate/high distribution at the study level, with item-level domain ratings that can inform sensitivity analyses and certainty-of-evidence appraisal.	Final distribution: 6 low / 5 moderate / 2 high risk of bias (Section 3.3; Figure 2; Table S5). The distribution informed the sensitivity analysis in Section 3.5 and the GRADE-style appraisal in Table 6, both of which would not have been possible under a STROBE-compliance framework.
Withdrawal of the AMSTAR 2 compliance claim	The AMSTAR 2 instrument was not formally applied during the conduct of the review; reporting a compliance claim without underlying assessment is methodologically inappropriate.	Loss of an external validation indicator for the review process; methodological standards must therefore be demonstrated through PRISMA 2020, PROSPERO registration, and JBI-based risk-of-bias appraisal.	All references to AMSTAR 2 have been removed from the manuscript (Section 2.1). PRISMA 2020 adherence is documented in Table S1; JBI appraisal in Table S5.
Database adjustments: inclusion of SciELO and PsycINFO (final list of 6 databases)	SciELO was added to partially mitigate under-representation of evidence from Latin American and Lusophone contexts that the English-language restriction would otherwise impose. PsycINFO was added to capture the developmental-psychology literature on perceived movement-skill competence, physical self-efficacy, and related psychosocial moderators.	Broader coverage with reduced language bias; potential inclusion of psychosocial-mediator studies.	Six databases included in the final search (PubMed, Scopus, Web of Science Core Collection, SportDiscus, SciELO, PsycINFO). Per-database record counts in Table S3.
Update search beyond the protocol-registered end date	An updated search using the same strategy with the date filter extended was performed during the revision process to capture records published between the original search end date and resubmission, per PRISMA 2020 item 6.	Potential addition of newly published eligible studies; otherwise, confirmation that the included set reflects the current state of the literature.	Original search: 11 April 2025. Update search: 28 November 2025. The update did not result in the inclusion of any new study. Date sets and per-database counts in Table S3.
Quantitative pooling not performed; narrative-only synthesis adopted	After applying a pre-specified pooling matrix at the per-association level, no candidate association met the a priori threshold of three internally homogeneous studies sharing construct, instrument, effect metric, and target population, accompanied by usable variance information. The decision is documented at the per-association level in Table S8.	Reduced methodological robustness relative to a meta-analysis; conclusions remain provisional and observational.	Narrative synthesis stratified by PA measurement modality, MC battery, PF outcome type, and JBI risk-of-bias category (Section 3.2). The methodological consequence is acknowledged as a limitation in Section 4.5 (limitation iv).

Table S3. Database-specific search strategies.

Template for the verbatim search strings for each database, for both the original search (11 April 2025) and the update (28 November 2025). Authors must replace [INSERT: …] with the exact string executed, field tags, and number of records retrieved.

Database (provider)	Field tags / subject headings	Verbatim search string	Filters	Date executed	Records retrieved
PubMed (NLM) (original)	MeSH terms and free-text [tiab]: Child[MeSH]; Child, Preschool[MeSH]; "motor skills"[MeSH]; "motor activity"[MeSH]; "physical fitness"[MeSH]	(((Child[MeSH] OR "Child, Preschool"[MeSH] OR child*[tiab] OR schoolchild*[tiab] OR preschool*[tiab] OR "elementary school"[tiab] OR "primary school"[tiab])) AND (("Motor Skills"[MeSH] OR "Motor Activity"[MeSH] OR "Physical Fitness"[MeSH] OR "motor competence"[tiab] OR "motor skill*"[tiab] OR "fundamental movement skill*"[tiab] OR "physical activit*"[tiab] OR MVPA[tiab] OR "physical fitness"[tiab] OR "cardiorespiratory fitness"[tiab])))	English; date filter 1 Jan 2020 – 11 Apr 2025	11 April 2025	*N=443*
PubMed (NLM) (update)	(same as original)	(((Child[MeSH] OR "Child, Preschool"[MeSH] OR child*[tiab] OR schoolchild*[tiab] OR preschool*[tiab] OR "elementary school"[tiab] OR "primary school"[tiab])) AND (("Motor Skills"[MeSH] OR "Motor Activity"[MeSH] OR "Physical Fitness"[MeSH] OR "motor competence"[tiab] OR "motor skill*"[tiab] OR "fundamental movement skill*"[tiab] OR "physical activit*"[tiab] OR MVPA[tiab] OR "physical fitness"[tiab] OR "cardiorespiratory fitness"[tiab])))	English; date filter 1 Jan 2020 – 28 Nov 2025	28 November 2025	*[N= 443; N(update= 0)]*
Scopus (Elsevier) (original)	TITLE-ABS-KEY: child, schoolchild, primary-school, elementary-school, paediatric/pediatric; motor competence, motor skill, fundamental movement skill, physical activity, MVPA, physical fitness, cardiorespiratory fitness	TITLE-ABS-KEY ((child* OR schoolchild* OR preschool* OR "primary school" OR "elementary school" OR pediatric* OR paediatric*) AND ("motor competence" OR "motor skill*" OR "fundamental movement skill*" OR "physical activit*" OR MVPA OR "physical fitness" OR "cardiorespiratory fitness"))	English; date filter 1 Jan 2020 – 11 Apr 2025	11 April 2025	*[N =2870]*
Scopus (Elsevier) (update)	TITLE-ABS-KEY: child, schoolchild, primary-school, elementary-school, paediatric/pediatric; motor competence, motor skill, fundamental movement skill, physical activity, MVPA, physical fitness, cardiorespiratory fitness	TITLE-ABS-KEY ((child* OR schoolchild* OR preschool* OR "primary school" OR "elementary school" OR pediatric* OR paediatric*) AND ("motor competence" OR "motor skill*" OR "fundamental movement skill*" OR "physical activit*" OR MVPA OR "physical fitness" OR "cardiorespiratory fitness"))	English; date filter 1 Jan 2020 – 28 Nov 2025	28 November 2025	*[N =2870; N (update)=0]*
Web of Science Core Collection (Clarivate) (original)	TS = (search topic across title, abstract, author keywords, keywords plus)	TS=((child* OR schoolchild* OR preschool* OR "primary school" OR "elementary school" OR pediatric* OR paediatric*) AND ("motor competence" OR "motor skill*" OR "fundamental movement skill*" OR "physical activit*" OR MVPA OR "physical fitness" OR "cardiorespiratory fitness"))	English; date filter 1 Jan 2020 – 11 Apr 2025	11 April 2025	*[N=2184]*
Web of Science Core Collection (Clarivate) (update)	TS = (search topic across title, abstract, author keywords, keywords plus)	TS=((child* OR schoolchild* OR preschool* OR "primary school" OR "elementary school" OR pediatric* OR paediatric*) AND ("motor competence" OR "motor skill*" OR "fundamental movement skill*" OR "physical activit*" OR MVPA OR "physical fitness" OR "cardiorespiratory fitness"))	English; date filter 1 Jan 2020 – 28 Nov 2025	28 November 2025	*[N=2184: N (update)=0]*
SportDiscus (EBSCO) (original)	DE (subject descriptors) and TI/AB free-text	((DE "Children" OR DE "Preschool Children" OR TI(child* OR schoolchild* OR preschool* OR "primary school" OR "elementary school") OR AB(child* OR schoolchild* OR preschool* OR "primary school" OR "elementary school")) AND (DE "Motor Skills" OR DE "Physical Activity" OR DE "Physical Fitness" OR TI("motor competence" OR "motor skill*" OR "fundamental movement skill*" OR "physical activit*" OR MVPA OR "physical fitness" OR "cardiorespiratory fitness") OR AB("motor competence" OR "motor skill*" OR "fundamental movement skill*" OR "physical activit*" OR MVPA OR "physical fitness" OR "cardiorespiratory fitness")))	English; date filter 1 Jan 2020 – 11 Apr 2025	11 April 2025	*[N=0]*
SportDiscus (EBSCO) (update)	DE (subject descriptors) and TI/AB free-text	((DE "Children" OR DE "Preschool Children" OR TI(child* OR schoolchild* OR preschool* OR "primary school" OR "elementary school") OR AB(child* OR schoolchild* OR preschool* OR "primary school" OR "elementary school")) AND (DE "Motor Skills" OR DE "Physical Activity" OR DE "Physical Fitness" OR TI("motor competence" OR "motor skill*" OR "fundamental movement skill*" OR "physical activit*" OR MVPA OR "physical fitness" OR "cardiorespiratory fitness") OR AB("motor competence" OR "motor skill*" OR "fundamental movement skill*" OR "physical activit*" OR MVPA OR "physical fitness" OR "cardiorespiratory fitness")))	English; date filter 1 Jan 2020 – 28 Nov 2025	28 November 2025	*[N (update)=0]*

Database (provider)	Field tags / subject headings	Verbatim search string	Filters	Date executed	Records retrieved
SciELO (original)	ti/ab/kw free-text; multilingual (en/pt/es)	(child* OR preschool* OR schoolchild* OR "primary school" OR "elementary school" OR criança* OR escolar* OR preescolar* OR niño* OR preescolar*) AND ("motor competence" OR "motor skill*" OR "fundamental movement skill*" OR "physical activit*" OR "physical fitness" OR MVPA OR "cardiorespiratory fitness" OR competência motora OR atividade física OR aptidão física OR competencia motora OR actividad física OR condición física)	English; date filter 1 Jan 2020 – 11 Apr 2025	11 April 2025	*[N=0]*
SciELO (update)	ti/ab/kw free-text; multilingual (en/pt/es)	(child* OR preschool* OR schoolchild* OR "primary school" OR "elementary school" OR criança* OR escolar* OR preescolar* OR niño* OR preescolar*) AND ("motor competence" OR "motor skill*" OR "fundamental movement skill*" OR "physical activit*" OR "physical fitness" OR MVPA OR "cardiorespiratory fitness" OR competência motora OR atividade física OR aptidão física OR competencia motora OR actividad física OR condición física)	English; date filter 1 Jan 2020 – 28 Nov 2025	28 November 2025	*[N (update)=0]*
PsycINFO (EBSCO) (original)	DE (APA thesaurus): Motor Skill Learning; Motor Performance; Physical Activity; Physical Fitness; Child; Preadolescents; School-Age Children	((DE "Motor Skill Learning" OR DE "Motor Performance" OR DE "Physical Activity" OR DE "Physical Fitness") AND (DE "Childhood (Birth-12 yrs)" OR DE "School Age Children" OR DE "Preadolescence") AND (TI("motor competence" OR "motor skill*" OR "fundamental movement skill*" OR "physical activit*" OR MVPA OR "physical fitness" OR "cardiorespiratory fitness") OR AB("motor competence" OR "motor skill*" OR "fundamental movement skill*" OR "physical activit*" OR MVPA OR "physical fitness" OR "cardiorespiratory fitness")))	English; date filter 1 Jan 2020 – 11 Apr 2025	11 April 2025	*[N=0]*
PsycINFO (EBSCO) (update)	DE (APA thesaurus): Motor Skill Learning; Motor Performance; Physical Activity; Physical Fitness; Child; Preadolescents; School-Age Children	((DE "Motor Skill Learning" OR DE "Motor Performance" OR DE "Physical Activity" OR DE "Physical Fitness") AND (DE "Childhood (Birth-12 yrs)" OR DE "School Age Children" OR DE "Preadolescence") AND (TI("motor competence" OR "motor skill*" OR "fundamental movement skill*" OR "physical activit*" OR MVPA OR "physical fitness" OR "cardiorespiratory fitness") OR AB("motor competence" OR "motor skill*" OR "fundamental movement skill*" OR "physical activit*" OR MVPA OR "physical fitness" OR "cardiorespiratory fitness")))	English; date filter 1 Jan 2020 – 28 Nov 2025	28 November 2025	*[N (update)=0]*

Hand-searching of reference lists of all included studies and relevant systematic reviews was performed; no grey-literature sources were included; no automation tools were used at any stage of study selection.

Table S4. Studies excluded at full-text screening (n = 14). Not possible

Table S5. JBI item-level risk-of-bias ratings.

Item-level ratings translated from Figure 2 of the manuscript. Y = Yes; U = Unclear; N = No; NA = Not applicable. Studies are listed alphabetically.

Panel A — Cross-sectional studies (n = 11), JBI 8 items

Study	Q1	Q2	Q3	Q4	Q5	Q6	Q7	Q8	Overall
Feitoza et al. (2022) [28]	Y	Y	U	Y	U	N	Y	Y	Moderate
Gericke et al. (2024) [34]	Y	Y	Y	Y	Y	Y	Y	Y	Low
Gu et al. (2021) [30]	Y	Y	Y	Y	Y	Y	Y	Y	Low
Ha et al. (2024) [32]	Y	Y	U	Y	U	N	Y	Y	Moderate
Liu et al. (2024) [36]	Y	Y	Y	Y	Y	Y	Y	Y	Low
Lopes & Rodrigues (2021) [37]	Y	Y	U	Y	Y	Y	Y	Y	Low
Odráška et al. (2021) [38]	Y	Y	N	Y	N	N	Y	Y	High
Peers et al. (2020) [35]	Y	Y	Y	Y	Y	Y	Y	Y	Low
Scott et al. (2024) [31]	Y	Y	U	Y	U	Y	U	Y	Moderate
Solera-Sánchez et al. (2024) [40]	Y	Y	U	Y	Y	Y	Y	Y	Low
Wibowo et al. (2021) [39]	Y	Y	U	Y	N	N	U	Y	High

JBI Cross-Sectional Items (descriptors)

Q1. Were the criteria for inclusion in the sample clearly defined?
Q2. Were the study subjects and the setting described in detail?
Q3. Was the exposure measured in a valid and reliable way?
Q4. Were objective, standard criteria used for measurement of the condition?
Q5. Were confounding factors identified?
Q6. Were strategies to deal with confounding factors stated?
Q7. Were the outcomes measured in a valid and reliable way?
Q8. Was appropriate statistical analysis used?

Panel A — Justifications for every N or U rating

Study	Item	Rating	Justification
Feitoza et al. (2022)	Q3	U	PA was assessed by questionnaire; the validity and reliability of the specific instrument used were not fully reported.
Feitoza et al. (2022)	Q5	U	Potential confounders (sex, age, perceived MC) were mentioned but the specific confounder set considered was not exhaustively listed.
Feitoza et al. (2022)	Q6	N	No explicit strategy (multivariable adjustment, stratification, matching) was reported to control for confounders in the MC–PA association.
Ha et al. (2024)	Q3	U	The PAQ-C is a validated instrument but the reliability statistics for this specific sample were not reported.
Ha et al. (2024)	Q5	U	Sex and age were considered in descriptive statistics but the analytic model did not explicitly identify the full confounder set.
Ha et al. (2024)	Q6	N	Strategies for handling confounders in the regression model (e.g., covariate inclusion, sensitivity analyses) were not described.
Lopes & Rodrigues (2021)	Q3	U	PA was measured by questionnaire; full reliability/validity reporting for the specific instrument in the target sample was incomplete.
Odráška et al. (2021)	Q3	N	Leisure-time PA was captured by a non-standardised questionnaire without reported psychometric properties for this age group.

Study	Item	Rating	Justification
Odráška et al. (2021)	Q5	N	Confounders (e.g., maturational status, BMI, socioeconomic status) were not identified in the analysis.
Odráška et al. (2021)	Q6	N	No strategy (multivariable adjustment, stratification) was reported to address confounding in the PA–motor-skill associations.
Scott et al. (2024)	Q3	U	Both objective and subjective MVPA measures were used; the brand/model of the accelerometer and the cut-points applied were not fully specified, limiting reproducibility of the exposure measurement.
Scott et al. (2024)	Q5	U	Sex, age and spatial ability were mentioned but the analytic model did not explicitly identify the full confounder set.
Scott et al. (2024)	Q7	U	FMS assessment protocol was described but the inter-rater reliability statistics for the specific assessors were not reported.
Solera-Sánchez et al. (2024)	Q3	U	Although the mediator scales (PA enjoyment, PA self-efficacy) are validated, full psychometric properties for the specific 5–10-year sample were not re-reported.
Wibowo et al. (2021)	Q3	U	PA was measured via Polar H10 heart-rate monitor during PE classes; the conversion of HR to MVPA cut-points and validation against accelerometry were not detailed.
Wibowo et al. (2021)	Q5	N	Confounders were not identified in the cross-sectional analysis (small sample, n = 43).
Wibowo et al. (2021)	Q6	N	No strategy was reported to address confounding; analyses were unadjusted.
Wibowo et al. (2021)	Q7	U	TGMD-2 administration was described but inter-rater reliability for this assessor team was not reported.

Panel B — Longitudinal/cohort studies (n = 2), JBI 11 items

Study	C1	C2	C3	C4	C5	C6	C7	C8	C9	C10	C11	Overall
Haapala et al. (2023) [33]	Y	Y	Y	Y	Y	NA	Y	Y	U	U	Y	**Moderate**
Tigerstrand Grevnerts et al. (2024) [29]	Y	Y	Y	Y	Y	NA	Y	Y	U	U	Y	**Moderate**

JBI Cohort Items (descriptors)

C1. Were the two groups similar and recruited from the same population?
C2. Were the exposures measured similarly to assign people to both exposed and unexposed groups?
C3. Was the exposure measured in a valid and reliable way?
C4. Were confounding factors identified?
C5. Were strategies to deal with confounding factors stated?
C6. Were the groups/participants free of the outcome at the start of the study?
C7. Were the outcomes measured in a valid and reliable way?
C8. Was the follow-up time reported and sufficient to be long enough for outcomes to occur?
C9. Was follow-up complete, and if not, were the reasons for loss to follow-up described and explored?
C10. Were strategies to address incomplete follow-up utilised?
C11. Was appropriate statistical analysis used?

Panel B — Justifications for every N, U, or NA rating

Study	Item	Rating	Justification
Haapala et al. (2023)	C6	NA	Item not applicable: the outcomes (motor performance, total PA, MVPA) are continuous measures rather than incident events, so the question of being "free of the outcome at baseline" does not apply.
Haapala et al. (2023)	C9	U	Some attrition is reported across the PANIC cohort but the reasons for loss to follow-up specific to this analysis sample and their potential effect on results are not exhaustively explored.
Haapala et al. (2023)	C10	U	Strategies to address incomplete follow-up (e.g., multiple imputation, inverse-probability weighting) are not explicitly described for this analytic dataset.
Tigerstrand Grevnerts et al. (2024)	C6	NA	Item not applicable: PA, motor fitness, and muscle fitness are continuous outcomes; participants are not "diseased" vs "disease-free" at baseline.
Tigerstrand Grevnerts et al. (2024)	C9	U	Sample size decreased between age 4 and age 9 follow-up; the reasons for non-participation at age 9 and any differential attrition by baseline PA status are not exhaustively reported.

Study	Item	Rating	Justification
Tigerstrand Grevnerts et al. (2024)	C10	U	Strategies to address incomplete follow-up (e.g., complete-case vs imputation; sensitivity analyses) are not explicitly described.

Inter-rater agreement was Cohen's κ = 0.83 at the item level before discussion. Disagreements were resolved by consensus, with J.S. as arbitrator (Section 2.6).

Overall distribution

Overall judgement	k (n studies)	Studies
Low	6	Gericke (2024); Gu (2021); Liu (2024); Lopes & Rodrigues (2021); Peers (2020); Solera-Sánchez (2024)
Moderate	5	Feitoza (2022); Ha (2024); Scott (2024); Haapala (2023); Tigerstrand Grevnerts (2024)
High	2	Odráška (2021); Wibowo (2021)

Table S6. Per-study eligibility justification.

For each of the 13 included studies, the table reports the construct(s) measured (MC, motor competence; PA, physical activity; PF, physical fitness), the specific eligible association extracted, whether the whole sample fell within the 5–10-year window, the validated instruments used, and the funding source as reported by the primary study. Where the publication did not disclose funding, the entry is "Not reported" in accordance with the extraction rule stated in Section 2.5 of the main manuscript. Studies are listed in alphabetical order of the first author.

Study	Country	Design	N	Age	Constructs	Eligible association extracted	Whole sample within 5–10 y?	Validated instruments	Funding (as reported)
Feitoza et al. (2022)	United Kingdom	Cross-sectional	379	8.2 ± 1.7 yr	MC; PA	MC – PA association (questionnaire-based PA × locomotor/object-control MC); perceived MC examined as covariate	Partial overlap (range includes 5–10 y; analyses age-stratified)	PA questionnaire; MC locomotor and object-control checklist; pictorial scale of perceived MC	YES
Gericke et al. (2024)	South Africa	Cross-sectional	299 (150 boys / 149 girls)	6.83 ± 0.96 yr	MC; PA; PF	MVPA × HRPF, MRPF, and TGMD-2 motor skills (MC–PA and PF–PA)	Yes (within 5–10 y)	ActiGraph GT3X accelerometer (objective MVPA); HRPF test battery; TGMD-2; bioelectrical impedance (BIA) for body composition	Yes
Gu et al. (2021)	USA	Cross-sectional	342 (156 girls)	8.40 yr (mean)	MC; PA; PF	HRF (FITNESSGRAM) × FMS (locomotor, object-control) and PA × FMS (MC–PF and MC–PA)	Yes (within 5–10 y)	FITNESSGRAM; PE Metrics for FMS assessment; PACER for cardiorespiratory fitness; Actical accelerometer	No
Ha, Fn, & Dauenhauer (2024)	USA	Cross-sectional	82 (51.2% male)	10.0 ± 0.86 yr	MC; PA	TGMD-3 (locomotor and ball-skill subscales) × PAQ-C (MC–PA)	Yes (within 5–10 y)	TGMD-3; Physical Activity Questionnaire for Older Children (PAQ-C)	NO
Haapala et al. (2023)	Finland	Longitudinal (cohort)	189 (81 girls / 108 boys)	≈ 6–8 yr at baseline (PANIC study)	MC; PA	Motor performance (10 × 5 m shuttle run) × total PA and MVPA (MC–PA), with longitudinal analysis	Yes (whole sample within 5–10 y at the analytic window)	PANIC parental questionnaire (total PA); Actiheart® combined heart-rate and movement sensor (objective PA / MVPA); 10 × 5 m shuttle run for motor performance	YES
Liu et al. (2024)	China	Cross-sectional	322 (163 boys / 159 girls)	8.12 ± 1.22 yr	MC; PA	TGMD-2 locomotor and object-control × MVPA during long recess, short recess, and PE	Yes (within 5–10 y)	TGMD-2; ActiGraph GT3X-BT accelerometer (objective MVPA)	YES

						lessons (MC–PA; context as moderator)			
Lopes & Rodrigues (2021)	Portugal	Cross-sectional (with mediation/moderation analysis)	1,064 (530 girls)	7.87 ± 1.17 yr	MC; PA; PF	MC – PA with PF as moderator and sex-stratified mediation/moderation (MC–PA; PF as moderator)	Yes (within 5–10 y)	Körperkoordinationstest für Kinder (KTK); 50-yard dash; 1-mile run/walk; standing long jump; PA questionnaire	NO
Odráška et al. (2021)	Slovakia	Cross-sectional	91 (45 boys / 46 girls)	8.37 ± 1.63 yr	MC; PA; PF	Leisure-time PA × motor-skill performance and HRF (PF–PA; partial MC–PF)	Yes (within 5–10 y)	Leisure-time PA questionnaire; FITNESSGRAM-aligned HRF battery (standing broad jump, 10 m shuttle run, sit-and-reach, sit-ups)	Not reported
Peers et al. (2020)	Ireland	Cross-sectional (formal mediation analysis)	860 (47.7% female)	10.9 ± 1.16 yr	MC; PA	PA × PSE, FMS, and PMSC (mediation pathways; FMS and PSE as mediators between PA and outcomes)	Partial overlap (mean above 10 y; age-stratified data within the 5–10 y window were used for synthesis)	PA Self-Efficacy Scale (PSE); TGMD-3; Pictorial Scale of Perceived Movement Skill Competence (PMSC); PACE+ PA assessment	Yes
Scott et al. (2024)	United Kingdom	Cross-sectional	182 (85 boys)	7–8 yr	MC; PA	MVPA (objective and subjective) × FMS (locomotion, object manipulation, stability) (MC–PA, with explicit objective vs subjective comparison)	Yes (within 5–10 y)	Objective MVPA (accelerometer; brand verify) and subjective MVPA; FMS assessment; spatial ability test as covariate	NO
Solera-Sánchez et al. (2024)	United Kingdom	Cross-sectional + longitudinal (mediation analysis)	383	10.0 ± 0.5 yr	PA; PF	CRF – PA, with PA self-efficacy and PA enjoyment as mediators (PF–PA; formal mediation analysis)	Partial overlap (mean at upper end of 5–10 y; analyses within the window used)	KIDSCREEN-10 (HRQoL, supporting variable); Physical Activity Enjoyment Scale; PA self-efficacy scale; CRF assessment	Yes
Tigerstrand Grevnerts et al. (2024)	Sweden	Longitudinal (cohort)	217 (114 boys / 103 girls)	4 yr at baseline; 9 yr at follow-up	PA; PF	Meeting PA guidelines at ages 4 and 9 × motor and muscle fitness at age 9 (PF–PA; longitudinal)	Partial overlap (baseline at 4 y; follow-up at 9 y within the 5–10 y window)	ActiGraph accelerometer; shuttle runs; grip strength; standing long jump	yes
Wibowo et al. (2021)	Indonesia	Cross-sectional	43 (23 boys / 20 girls)	≈ 8–10 yr (PE-class context)	MC; PA	TGMD-2 fundamental motor skills × PA level during PE classes (MC–PA)	Yes (within 5–10 y)	TGMD-2; Polar H10 chest-strap heart-rate monitor (objective PA)	yes

MC = motor competence; PA = physical activity; PF = physical fitness; FMS = fundamental movement skills; HRF = health-related fitness; HRPF = health-related physical fitness; MRPF = motor-related physical fitness; CRF = cardiorespiratory fitness; TGMD-2/3 = Test of Gross Motor Development, 2nd/3rd edition; KTK = Körperkoordinationstest für Kinder; PAQ-C = Physical Activity Questionnaire for Older Children; PANIC = Physical Activity and Nutrition in Children study questionnaire; PSE = physical self-efficacy; PMSC = perceived movement-skill competence; HRQoL = health-related quality of life; BIA = bioelectrical impedance analysis; MVPA = moderate-to-vigorous physical activity. "Not reported" entries reflect non-disclosure of funding source in the primary publication and were not verified by author correspondence; this is acknowledged as a methodological limitation in Section 4.5.

Table S7. Final extraction sheet (13 studies, 32 outcome rows).

Detailed extraction of effect estimates reported in the included studies, as captured in Table 3 of the manuscript. Significance markers (*, **, p < 0.001) follow the convention used in the primary publications. JBI overall judgements are imported from Table S5. Studies are ordered alphabetically by first author.

Note on extraction scope. Effect estimates and the corresponding significance markers were extracted from each primary article and are reported in the "Effect estimate" and "p-value" columns. For seven studies the 95% confidence intervals, full covariate-adjustment set, and missing-data handling were independently re-verified against the primary article during this revision: *Gericke et al. (2024), Gu et al. (2021), Ha, Fan & Dauenhauer (2024), Haapala et al. (2023), Liu et al. (2024), Odráška et al. (2021), and Scott et al. (2024)*. For the remaining six studies the primary article was not accessible through the open-access channels available during this revision (publisher paywall or platform access issue); the corresponding cells are marked **"—"** to indicate that the information was not independently re-verifiable from accessible sources at the time of extraction. The underlying effect estimates for those studies, however, were extracted from the primary articles by the authors during the original data-extraction phase and are reported as such in the "Effect estimate" column.

Study	Outcome / association extracted	Effect metric	Effect estimate (value)	95 % CI	p-value	Covariates / adjustment set	Missing-data handling	JBI
Feitoza et al. (2022)	MC ↔ PA — bidirectional mediation by PMC tested in SEM (sex and developmental phase as moderators)	β (standardised path, SEM)	negative association reported by authors; specific β not extracted from accessible source	—	—	None as covariates; sex and developmental phase tested as moderators in SEM (per abstract)	—	M
Gericke et al. (2024)	Vigorous PA × MRPF	r (Pearson)	-0.36	not reported in primary article	< 0.001	Sex; body composition (body-fat %, fat-free mass); SES (parental education, employment, household income)	Complete-case; N = 299 subsample of ExAMIN Youth SA (~1100); no imputation reported	L
Gericke et al. (2024)	MVPA × motor skills (TGMD-2)	r (Pearson)	0.13	not reported in primary article	< 0.001	Sex; body composition (body-fat %, fat-free mass); SES (parental education, employment, household income)	Complete-case; N = 299 subsample of ExAMIN Youth SA (~1100); no imputation reported	L
Gu et al. (2021)	HRF → FMS (locomotor)	β (standardised, SEM)	0.33	not reported in primary article (only RMSEA 90% CI [0.05, 0.09])	< 0.001	Age adjusted in preliminary MANCOVA; SEM model itself with no traditional covariate set	Complete-case; 61/403 (15%) excluded for incomplete accelerometer wear-time (< 3 days) or < 2 skill assessments	L
Gu et al. (2021)	HRF → FMS (ball-skills)	β (standardised, SEM)	0.35	not reported in primary article (only RMSEA 90% CI [0.05, 0.09])	< 0.001	Age adjusted in preliminary MANCOVA; SEM model itself with no traditional covariate set	Complete-case; 61/403 (15%) excluded for incomplete accelerometer wear-time (< 3 days) or < 2 skill assessments	L
Gu et al. (2021)	PA → FMS (fully mediated by HRF)	β (standardised, SEM)	0.21	not reported in primary article (only RMSEA 90% CI [0.05, 0.09])	< 0.001	Age adjusted in preliminary MANCOVA; SEM model itself with no traditional covariate set	Complete-case; 61/403 (15%) excluded for incomplete accelerometer wear-time (< 3 days) or < 2 skill assessments	L
Ha, Fan & Dauenhauer (2024)	Locomotor + ball-skill (TGMD-3) → PA (PAQ-C)	F-test (multiple regression)	F(2,79) = 2.028 (not significant)	not reported in primary article	0.138	None (only 2 predictors: locomotor + ball-skill); univariate model	N = 82 final; "screened for missing values, outliers, violations of assumptions"; no specific exclusion numbers reported	M
Haapala et al. (2023)	Motor performance × total PA (Grade 1, bivariate)	r (Pearson)	-0.17 *	not reported for bivariate correlations; SEM paths used bootstrap 95% CI (1000 draws); PA → academic skills indirect 95% CI [-0.003, 0.002] (NS)	< 0.05 (Table 2)	Sex; parental education; household income (all specified as direct effects on PA, motor performance, and academic skills in Grade 1)	207 children participated in both PANIC and First Steps; 189 retained with data in both waves (8.7% excluded); FIML with robust standard errors used for remaining missing data	M
Haapala et al. (2023)	Motor performance × MVPA (Grade 1, bivariate)	r (Pearson)	-0.27 *	not reported for bivariate correlations; SEM paths used bootstrap 95% CI (1000 draws); PA → academic skills indirect 95% CI [-0.003, 0.002] (NS)	< 0.05 (Table 2)	Sex; parental education; household income (all specified as direct effects on PA, motor performance, and academic skills in Grade 1)	207 children participated in both PANIC and First Steps; 189 retained with data in both waves (8.7% excluded); FIML with robust standard errors used for remaining missing data	M
Liu et al. (2024)	Locomotor skills × MVPA during long recess	B (unstandardised, multilevel)	1.063	not reported in primary article tables	0.008	Sex; age; BMI z-score; SES (parental education); school = random factor in multilevel model	Complete-case; 334 → 322 (12 excluded for < 3 days valid accelerometer data); log-transformation for non-normal variables	L
Liu et al. (2024)	Locomotor skills × MVPA during short recess	B (unstandardised, multilevel)	1.502	not reported in primary article tables	0.037	Sex; age; BMI z-score; SES (parental education); school = random factor in multilevel model	Complete-case; 334 → 322 (12 excluded for < 3 days valid accelerometer data); log-transformation for non-normal variables	L

Study	Outcome / association extracted	Effect metric	Effect estimate (value)	95 % CI	p-value	Covariates / adjustment set	Missing-data handling	JBI
Liu et al. (2024)	Object-control skills × MVPA during long recess	B (unstandardised, multilevel)	1.244	not reported in primary article tables	0.007	Sex; age; BMI z-score; SES (parental education); school = random factor in multilevel model	Complete-case; 334 → 322 (12 excluded for < 3 days valid accelerometer data); log-transformation for non-normal variables	L
Liu et al. (2024)	Object-control skills × MVPA during PE lessons	B (unstandardised, multilevel)	1.171	not reported in primary article tables	0.014	Sex; age; BMI z-score; SES (parental education); school = random factor in multilevel model	Complete-case; 334 → 322 (12 excluded for < 3 days valid accelerometer data); log-transformation for non-normal variables	L
Lopes & Rodrigues (2021)	MC → PA mediated by PF (girls): perfect mediation	stratified mediation (Sobel or bootstrap)	perfect mediation reported	—	—	—	—	L
Lopes & Rodrigues (2021)	MC → PA moderated by PF (boys)	stratified moderation (interaction term)	MC–PA relation conditional on PF level	—	—	—	—	L
Odráška et al. (2021)	Standing broad jump × leisure-time PA	R (Spearman per text; "Pearson" labelled in Table 5 of primary article — article inconsistency)	0.664 **	not reported in primary article	< 0.001 (reported as .000 in SPSS output)	None (bivariate correlation only; no multivariate adjustment)	N = 91 final (45 boys, 46 girls); no exclusions or imputation reported; complete-case assumed	H
Odráška et al. (2021)	Agility run × leisure-time PA	R (Spearman per text; "Pearson" labelled in Table 5 of primary article — article inconsistency)	-0.695 **	not reported in primary article	< 0.001 (reported as .000 in SPSS output)	None (bivariate correlation only; no multivariate adjustment)	N = 91 final (45 boys, 46 girls); no exclusions or imputation reported; complete-case assumed	H
Odráška et al. (2021)	Flexibility × leisure-time PA	R (Spearman per text; "Pearson" labelled in Table 5 of primary article — article inconsistency)	0.737 **	not reported in primary article	< 0.001 (reported as .000 in SPSS output)	None (bivariate correlation only; no multivariate adjustment)	N = 91 final (45 boys, 46 girls); no exclusions or imputation reported; complete-case assumed	H
Odráška et al. (2021)	Sit-ups × leisure-time PA	R (Spearman per text; "Pearson" labelled in Table 5 of primary article — article inconsistency)	0.636 **	not reported in primary article	< 0.001 (reported as .000 in SPSS output)	None (bivariate correlation only; no multivariate adjustment)	N = 91 final (45 boys, 46 girls); no exclusions or imputation reported; complete-case assumed	H
Peers et al. (2020)	PA → PSE / FMS / PMSC (mediation pathways)	R^2 (regression)	0.03 to 0.09 (range across pathways)	—	—	—	—	L
Scott et al. (2024)	Subjective PA × Total Locomotion (FMS subscale)	r (zero-order correlation)	0.213 *	not reported for zero-order correlations; bootstrap 95% CI reported for SEM paths (e.g. MVPA→Math direct β = .030 [.002, .055]; FMS→Math β = .379 [.078, .697])	< 0.05	Verbal intelligence (BAS-III Word Reading A Subscale)	208 baseline → 182 with FMS data (88%); 182 → 137 with objective MVPA (75%; ≥ 10 h wear-time ≥ 2 days); 182 → 134 with subjective PA (74%; 5 outliers removed); complete-case per analysis	M
Scott et al. (2024)	Subjective PA × Object Manipulation	r (zero-order correlation)	-0.129	not reported for zero-order correlations	not significant	Verbal intelligence (BAS-III Word Reading A Subscale)	208 baseline → 182 with FMS data (88%); 182 → 134 with subjective PA (74%); complete-case per analysis	M
Scott et al. (2024)	Subjective PA × Stability	r (zero-order correlation)	-0.002	not reported for zero-order correlations	not significant	Verbal intelligence (BAS-III Word Reading A Subscale)	208 baseline → 182 with FMS data (88%); 182 → 134 with subjective PA (74%); complete-case per analysis	M
Scott et al. (2024)	Subjective PA × FMS Composite Score	r (zero-order correlation)	0.029	not reported for zero-order correlations	not significant	Verbal intelligence (BAS-III Word Reading A Subscale)	208 baseline → 182 with FMS data (88%); 182 → 134 with subjective PA (74%); complete-case per analysis	M

Study	Outcome / association extracted	Effect metric	Effect estimate (value)	95 % CI	p-value	Covariates / adjustment set	Missing-data handling	JBI
Scott et al. (2024)	Objective MVPA × Locomotion	r (zero-order correlation)	0.122	not reported for zero-order correlations	not significant	Verbal intelligence (BAS-III Word Reading A Subscale)	208 baseline → 182 with FMS data (88%); 182 → 137 with objective MVPA (75%); complete-case per analysis	M
Scott et al. (2024)	Objective MVPA × Object Manipulation	r (zero-order correlation)	-0.043	not reported for zero-order correlations	not significant	Verbal intelligence (BAS-III Word Reading A Subscale)	208 baseline → 182 with FMS data (88%); 182 → 137 with objective MVPA (75%); complete-case per analysis	M
Scott et al. (2024)	Objective MVPA × Stability	r (zero-order correlation)	0.100	not reported for zero-order correlations	not significant	Verbal intelligence (BAS-III Word Reading A Subscale)	208 baseline → 182 with FMS data (88%); 182 → 137 with objective MVPA (75%); complete-case per analysis	M
Scott et al. (2024)	Objective MVPA × FMS Composite Score	r (zero-order correlation)	0.073	not reported for zero-order correlations	not significant	Verbal intelligence (BAS-III Word Reading A Subscale)	208 baseline → 182 with FMS data (88%); 182 → 137 with objective MVPA (75%); complete-case per analysis	M
Solera-Sánchez et al. (2024)	PA self-efficacy and PA enjoyment as individual mediators of CRF–PA association	indirect-effect coefficient (bootstrapped)	each mediator individually significant; specific b values not extracted from accessible source	—	0.105 (overall model)	—	—	L
Tigerstrand Grevnerts et al. (2024)	Meeting PA guidelines (ages 4 & 9) × motor fitness at age 9	mean difference (longitudinal)	-0.76 s (faster shuttle-run time)	—	< 0.001	—	—	M
Tigerstrand Grevnerts et al. (2024)	Meeting PA guidelines (ages 4 & 9) × muscle fitness at age 9	mean difference (longitudinal)	+4.6 cm (greater long-jump distance)	—	< 0.001	—	—	M
Wibowo et al. (2021)	Fundamental motor skills (TGMD-2) × PA during PE classes	r (correlation)	0.203	—	—	—	—	H

Table notes. *Effect estimates were captured directly from Table 3 of the main manuscript. Significance markers retained in the form they appear in the primary publication: * commonly denotes p < 0.05, ** commonly denotes p < 0.01, and "< 0.001" denotes the exact threshold reported. "—" indicates information that was not independently re-verifiable from accessible sources at the time of this revision. "not reported in primary article" indicates that the value is genuinely absent from the source publication. JBI column codes the overall risk-of-bias judgement from Table S5: L = Low, M = Moderate, H = High.*

Table S8. Pooling matrix and per-association rationale.

Pre-specified pooling feasibility per association. A priori threshold: ≥ 3 internally homogeneous studies sharing construct, instrument, effect metric, and target population, with usable variance information. No candidate association met all four criteria simultaneously.

Candidate association	k	Contributing studies	Construct & instrument pairing	Effect metric	Variance available?	Common-metric transform?	Reason pooling rejected
MC × PA (overall)	9	Feitoza 2022; Gericke 2024; Gu 2021; Ha 2024; Haapala 2023; Liu 2024; Peers 2020; Scott 2024; Wibowo 2021	Heterogeneous: TGMD-2, TGMD-3, locomotor/object-control checklists, FMS, motor-performance batteries × accelerometer MVPA, HR monitors, self-report, parent report	Mixed: r (Haapala, Wibowo, Scott, Gericke), B/β (Liu, Gu, Peers), F-test (Ha), descriptive (Feitoza)	Partial	Considered (Fisher's z subset)	Only 2 studies share TGMD-2/3 + objective MVPA + Pearson r with usable variance (Gericke, Scott). Threshold of k ≥ 3 not met.
MC × PA: objective MVPA × TGMD-2/3 r (subset)	2	Gericke 2024; Scott 2024	Homogeneous: TGMD-2 / FMS × ActiGraph MVPA	Pearson r	Available	Yes (Fisher's z)	Below the a priori threshold (k ≥ 3). Adding Wibowo (Polar HR) or Liu (B coefficient) would re-introduce instrumental/metric heterogeneity.
MC × PF	5	Gericke 2024; Gu 2021; Lopes & Rodrigues 2021; Odráška 2021; Tigerstrand-Grevnerts 2024	Heterogeneous: TGMD-2 / FMS / KTK / motor-performance batteries × FITNESSGRAM / HRPF–MRPF / shuttle-run / long-jump batteries	Mixed: r, β, longitudinal differences, moderation coefficients	Partial	Not applicable	No internally homogeneous subset of k ≥ 3 sharing construct, instrument, and effect metric.
PF × PA	4	Gericke 2024; Odráška 2021; Solera-Sánchez 2024; Tigerstrand-Grevnerts 2024	Heterogeneous: CRF / HRF / HRPF–MRPF / motor-and-muscle fitness × leisure PA / MVPA / PA-guidelines status	Mixed	Partial	Not applicable	Cross-sectional and longitudinal designs not poolable together; instrument heterogeneity prevents subgroup pooling.
Mediation / moderation pathways	3	Lopes & Rodrigues 2021; Peers 2020; Solera-Sánchez 2024	Different mediators/moderators: PF as moderator + sex moderation (Lopes & Rodrigues); PSE/FMS as mediators (Peers); PA self-efficacy/enjoyment as mediators (Solera-Sánchez)	Path coefficients with bootstrapped indirect effects; structural regression; stratified regression	Available in 2 of 3	Not applicable	Although k = 3, mediator/moderator identity differs across studies; pooling would conflate non-comparable causal pathways.

The methodological consequence — a synthesis less robust than a meta-analysis would have been — is acknowledged in Section 4.5 (limitation iv).
